# liputils: a Python module to manage individual fatty acid moieties from complex lipids

**DOI:** 10.1038/s41598-020-70259-9

**Published:** 2020-08-07

**Authors:** Stefano Manzini, Marco Busnelli, Alice Colombo, Mostafa Kiamehr, Giulia Chiesa

**Affiliations:** 1 Department of Pharmacological and Biomolecular Sciences, Università Degli Studi Di Milano, Milan, Italy; 2 Faculty of Medicine and Health Technology, Tampere University, 33014 Tampere, Finland

**Keywords:** Data processing, Software, Lipidomics

## Abstract

Lipidomic analyses address the problem of characterizing the lipid components of given cells, tissues and organisms by means of chromatographic separations coupled to high-resolution, tandem mass spectrometry analyses. A number of software tools have been developed to help in the daunting task of mass spectrometry signal processing and cleaning, peak analysis and compound identification, and a typical finished lipidomic dataset contains hundreds to thousands of individual molecular lipid species. To provide researchers without a specific technical expertise in mass spectrometry the possibility of broadening the exploration of lipidomic datasets, we have developed liputils, a Python module that specializes in the extraction of fatty acid moieties from individual molecular lipids. There is no prerequisite data format, as liputils extracts residues from RefMet-compliant textual identifiers and from annotations of other commercially available services. We provide three examples of real-world data processing with liputils, as well as a detailed protocol on how to readily process an existing dataset that can be followed with basic informatics skills.

## Introduction

Lipidomics is the analysis of the large-scale identification and quantification of individual lipid species, which relies—at its finest—on the analytical technology of chromatographic separation coupled to mass spectrometry (MS) and dedicated software^1,2^. Lipidomics is among the most juvenile—omic technologies, yet recent advances in MS, lipid biochemistry and software pipelines allow for high-throughput, unbiased analysis of diverse biomolecules. A typical workflow of a high-resolution, high-throughput lipidomics involves MS-based studies of the structure, composition, and quantity of lipids in biological systems—typically organs, cells, and bodily fluids. Analytes are then identified and quantified with software tools that can process MS data in an automated or semi-automated manner^3,4^. Most of the tools that are available on dedicated portals, such as LIPID MAPS^5^, are designed to tackle the problems such as fragments recognition, lipids reconstruction from fragment analysis and artefactual signals removal^4^. As of beginning of 2020, ~ 3,000 papers featuring “lipidomics” and “mass spectrometry” could be retrieved from Web of Science, showing an ever growing trend since 2003^6^.

A modern lipidomic analysis can contain hundreds of individual molecular lipid species, of the 43,645 annotated in databases such as LIPID MAPS, as of March 2020^5^. Lipids—generically defined as water-insoluble molecules, or molecules soluble in organic solvents—have been grouped into eight categories: fatty acyls (9,374), glycerolipids (7,608), glycerophospholipids (9,918), sphingolipids (4,438), sterol lipids (2,828), prenol lipids (1,353), saccharolipids (1,316) and polyketides (6,810), further divided into sub-categories^7^.

In living organisms, they are used as energy reserve^8^, structural components of biological membranes, transporters, lipoprotein components^9,10^, signalling molecules^11^ and inflammation regulators^12–14^. Recently, an ever-growing number of studies has searched the lipidome of bodily fluids for lipid biomarkers as proxies of disease state and risk stratification, nowadays employed for neurological diseases^15^, cancer^16^, atherosclerosis^17^ and countless other fields^18^.

Often, lipidomic research pinpoints to few, selected individual molecular lipids—or ratios thereof—the biological take home message of an experimental design or a biomarker signature^19^. This might lead to the inability to see the forest for the trees, as global changes in the lipidome can be overlooked. This is especially true considering that individual fatty acid moieties can be shuttled back and forth from different lipid classes by the extraordinarily complex and variegated enzymatic machinery that orchestrate the lipid metabolism.

We have developed liputils, a Python module to automate the extraction of fatty acid moieties from lipidomic data analytes, as an add-on of a complete lipidomic workflow. With liputils, it is possible to neatly reprocess lipidomic data to extract from the analyte annotation the information about the fatty residues contained in each sample, or further adapt the module to individual needs.

Herein, we give an overview of the module capabilities, including a practical step-by-step tutorial. Further, insights gained by reprocessing publicly available, real-world data, are presented.

## Materials and methods

### Software

Data was processed with SciPy (version 1.3.1)^20^, Numpy (version 1.15.4)^21^, and Pandas (version 0.23.4)^22^. Tabular data images were made with Libre Office’s Calc (https://www.libreoffice.org/). Data visualization was performed with matplotlib (version 3.0.2)^23^ and seaborn (version 0.9.0)^24^ libraries for the Python programming language. Principal Component Analysis (PCA) was performed with Scikit-learn (version 0.20.0)^25^. liputils can be automatically installed via pip, the Python package installer (as detailed in “Procedure”), or downloaded from GitHub (https://github.com/Stemanz/liputils/).

### Statistical analyses

Statistical analyses are detailed for each individual analysis in the appropriate figure or table caption. Multiple group comparisons were tested for statistical significance with ANOVA and Tukey post-hoc test built-in functions of the R software environment (version 3.6.1)^26^.

### Software setup

A working Python 3.6+ environment with packages from the SciPy ecosystem (https://www.scipy.org/) is a prerequisite for the use of liputils. To avoid to manually install all libraries cited throughout the tutorial, the open-source Python distribution oriented towards data science, such as Anaconda (https://www.anaconda.com/distribution/), already comes with all needed libraries and has pre-built binaries for Windows, macOS and GNU/Linux. All individual packages are available via the Pip Python Package Manager. This distribution will be used in the following tutorial.

### Alternative analysis packages

Similar features can be found in other freely available software suites. Lipid Mini-On^27^ is graphical user interface based tool for enrichment analysis of lipidomic data. The underlying rodin R package can recognize LipidMaps-compliant lipid identifiers, and parse chain information. A similar functionality is also provided by the name module of the lipyd Python library (https://saezlab.github.io/lipyd/). This module is part of a much larger software suite, primarily aimed at the preprocessing of raw MS data and metabolite identification from mass spectra. Conversely, liputils is a lightweight Python library in active development, much easier to install and use, that specifically focuses on the parsing of the lipid identifiers. While providing a similar carbon chains recognition feature that is found in the aforementioned packages, liputils can also recognize standardized lipid names, such as “arachidonic acid” or “linoleyl palmitate”, even if lipyd.name does offer a basic “greek names” recognition feature. Furthermore, liputils can handle mass isobars (see Supplemental Materials and Methods). Finally, it is possible to process a whole dataset into carbon chains information with a one-liner function call, by following the final steps of the Procedure described below.

### Procedure

The following protocol illustrates how to create an environment to use liputils, as well as the required steps to process published data. An active internet connection is required to download software and data. Further detailed information on additional features of liputils can be found at the project’s page (https://pypi.org/project/liputils/).

The steps used in this protocol are intended for machines running macOS or GNU/Linux, but they can also be followed by Windows users by running the protocol in the Windows Subsystem for Linux (WLS) or adapted to be run in a pure Windows environment. Even if liputils can be directly used from the CPython interpreter, the use of Jupyter notebooks (https://jupyter.org/) is strongly encouraged.

Some commands need to be executed from the UNIX shell (the Terminal app) and are prefixed by the “$” symbol. Python commands that need to be run in the CPython interpreter or in Jupyter Notebook are prefixed by “ ⋙ ”. These symbols shall not be inputted with the commands.

Open the UNIX shell. On macOS, this is done by running the Terminal app from the Utilities (to access Utilities, from Finder ⌘ + ⇧ + U, or Go > Utilities).

Create a folder to store the tutorial files. This can either be done from the operating system graphical user interface or in the command line. In this example we will create the folder “tutorial” on the desktop from the command line, then move into that folder:

- $ mkdir $HOME/Desktop/tutorial
- $ cd $HOME/Desktop/tutorial

Alternatively, it is possible to create a folder, then right click on it and choose Services > New Terminal at Folder in macOS. If that is not possible, this option needs to be enabled in System Preferences > Keyboard > Shortcuts > Services.

Create a new Python virtual environment named “liptest” to avoid affecting other projects in the machine. This is an optional step.

- $ conda create –name liptest python=3.7
- $ conda activate liptest

To install liputils, alongside all dependencies needed to run it, type

- $ pip install liputils

As an alternative to the above procedure—especially for machines that cannot rely on the availability of Python distributions that already pack scientific libraries—it is possible to manually install all the libraries required to follow this tutorial. For example, the following protocol sets up an appropriate environment in the Debian-based Raspberry Pi OS:

- $ python3 -m venv liptest
- $ source liptest/bin/activat
- $ pip3 install liputils
- $ pip3 install jupyter

Download the Supplementary Information^28^ that will be processed. Either use the browser to download the Excel table from the publisher’s website, or type in the command line:

- $ wget https://static-content.springer.com/esm/art%3A10.1038%2Fsrep44503/MediaObjects/41598_2017_BFsrep44503_MOESM67_ESM.xls

Last, run Jupyter Notebook to start the analysis. This will open up a new browser tab containing a basic file manager.

- $ jupyter notebook

Once in the browser, select New▾ > Python 3. This will open up a new tab, where it is possible to execute Python code.

The data table to be processed has one sample per column, and one lipid analyte per row (Supplementary Fig. S1A). liputils contains a function that can be called to automatically extract residue information from the table and package everything into a new table. The function expects that the input data table has lipid names in the first column, samples in the remainder columns, and only one row of headers. It is possible to pre-process the table in a spreadsheet, or load it with the appropriate parameters in pandas, by skipping the first two rows and row-indexing the second column. Run each command with ⏎ in CPython and ⇧ + ⏎ in Jupyter Notebook.

- ⋙ import pandas as pd
- ⋙ df = pd.read_excel("41598_2017_BFsrep44503_MOESM67_ESM.xls", index_col=1, header=2)

These commands will load the input table into the df object. Now it is possible to extract residue information from each lipid with the make_residues_table() function, that wraps most of the functionality of liputil’s Lipid class, for all samples:

-  ⋙ from liputils import make_residues_table
-  ⋙ res = make_residues_table(df, liptype=one)

These commands will produce the results table, stored in the res object. As the input table is not RefMet-compliant, it is necessary to call the function with the liptype = None parameter as the function defaults to RefMet nomenclature. When working with RefMet compliant data, omit the parameter. The results table can be now be written on disk:

-  ⋙ res.to_csv("Processed data.csv", sep=“\t")

This saves the results table in a .csv file, with tabulation character as spacing (Supplementary Fig. S1B). This file can be further edited with a spreadsheet software.

Note that make_residues_table() drops any non-numerical column in the input table. This is done to facilitate the processing, as leftover labels or other non-numerical information would necessarily have to be manually discarded, but also because it would be otherwise impossible to extract meaningful information. Please do check if all the columns from which residue data needs to be extracted have been interpreted by pandas as numerical in type.

### Use of experimental animals, and human participants

We confirm that no animal model or sample was used or obtained within the present study. No human data was also generated or obtained within the present study.

The only protocols described in the paper are software protocols. All data was retrieved from previously published papers or from online data banks; all these sources are not related to the present study.

## Results and discussion

**Figure 1:**
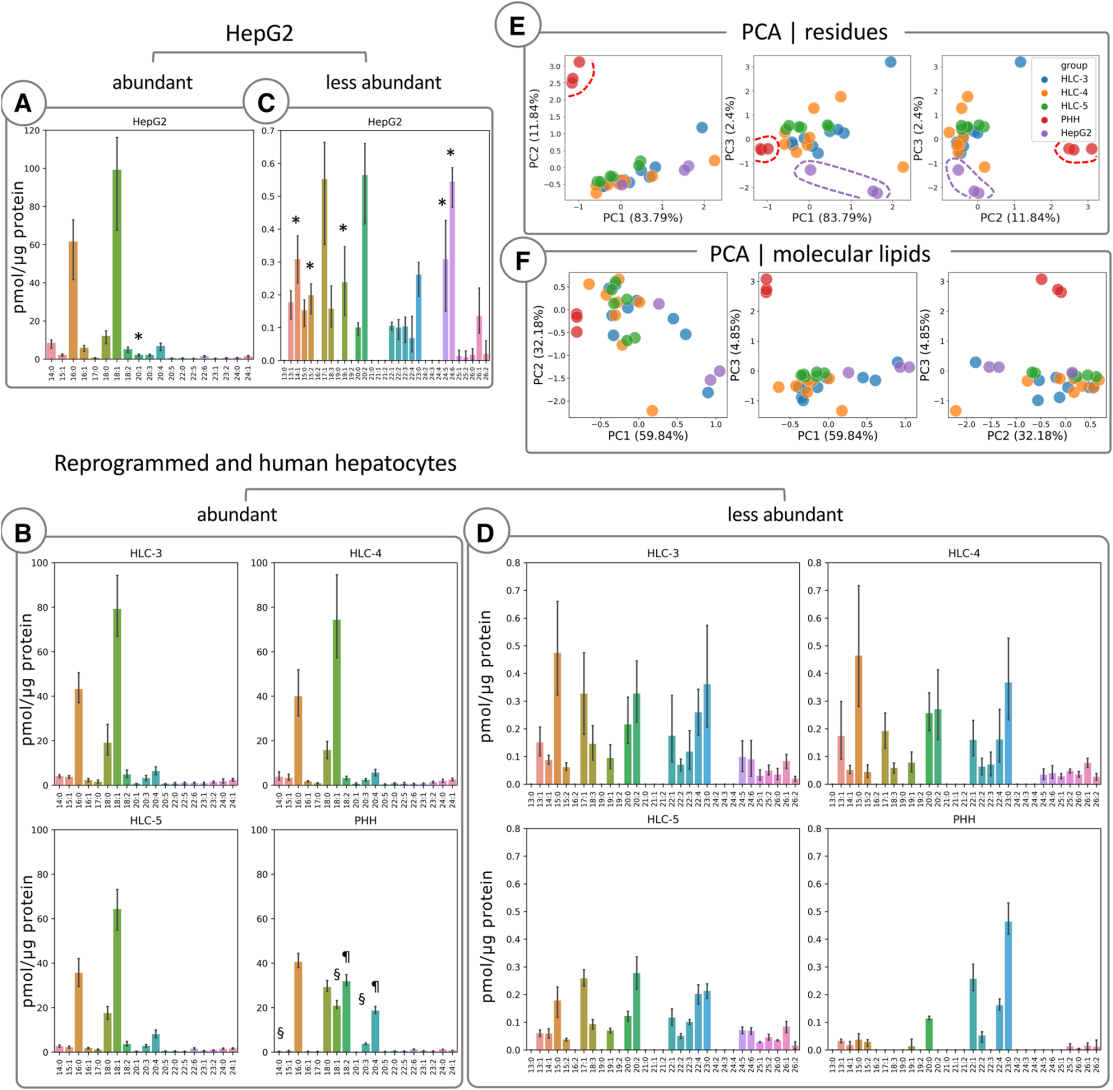
Lipidomics data from cultured primary hepatocytes, reprogrammed hepatocytes and HepG2 cells is reprocessed with liputils. Residues extracted from lipidomic data of HepG2, human hepatocyte-like cells (HLCs) and human primary hepatocytes (HPPs) are shown (**A**–**D**, n = 9 HLC-3, n = 8 HLC-4, n = 6 HLC-5, n = 3 PHH and HepG2 samples). Units were unchanged during the extraction and are the same as the input data (pmol/ μg protein). *p < 0.05 vs PHH, HLC-3, HLC-4 and HLC-5; ^§^p < 0.01 vs HepG2, HLC-3 and HLC-4; ^¶^p < 0.0001 vs HepG2, HLC-3, HLC-4 and HLC5. * indicates the similarities between PHHs and HLCs vs HepG2; ¶ and § indicate the similarities between HepG2 and HLCs vs PHHs. Statistically significant differences were determined with ANOVA followed by Tukey’s post-hoc test. Detailed statistics can be found in Supplementary Table S1. Histograms show the average ± SD. Scatter plots of the first three principal components (PCs) are shown for residues of each sample (**E**) and for molecular lipids for each sample (**F**).

We have developed a Python library, called liputils, to provide researchers a way to extract useful information from processed, final lipidomic data. Information—especially the number and type of fatty acid residues that are attached to a particular backbone—is obtained by text-based processing of the lipid identifiers.

liputils was designed to work with RefMet-compliant lipid nomenclature, and it is able to extract residues from either the official name or the common name of the analyte. For instance, “octadecatrienoic acid”, “linolenic acid” or FA(18:3) will all yield one residue of 18:3. Complex common names are decoded as well, “linoleyl palmitate” will yield one 16:0 and one 18:1 residue. Fully saturated, as well as mono- and polyunsaturated alkenyl fatty acids are supported. In case of unresolved ambiguities, such as in the case of the lipid PG(P-22:1/18:1)/PG(O-22:2/18:1), the default behaviour of liputils is reporting all residues 22:1, 18:1, 22:2 and 18:1, together with an index of 2 (in case of unambiguous results, the returned index is 1). When counting residues, liputils divides the number of times a residue is found, or the amount thereof, by the index. By this way, it is possible to correctly assign one 18:1 residue to that lipid and give an equal chance to the other two mass isobars (half the amount will be counted for each, or each will be counted 0.5 times). If this behaviour is not suited for the analysis, it is possible to discard all ambiguous mass isobars altogether while retaining residues from non-ambiguous lipids.

liputils is especially useful to extract broad residue signatures from high-resolution lipidomic data. To this aim, by using liputils, we reprocessed data from primary human hepatocytes (PHHs), HepG2 cells and hepatocyte-like cells (HLCs) obtained with three differentiation protocols from human induced pluripotent stem cells (iPSCs)^29,30^, and their culture media (Fig. 1, Supplementary Fig. S2)^31^. Every cell type revealed distinct residue patterns (Fig. 1). For abundant residues, above 0.5 pmol/μg, HLC and HepG2 shared similarities in the abundance of 18:2, 20:1 and 20:4 residues, (Fig. 1A,B). PHHs had higher levels of 18:2 and 20:4, and negligible 20:1 levels. Conversely, HLCs were much closer to PHHs with respect to 16:0 and 18:1 residues, higher in HepG2, even though the trend was not statistically significant. In less abundant residues (below 0.5 pmol/μg), HLCs and HepG2 showed a richer residue diversity compared to PHHs, even if HLCs had lower residue levels, finding a position midway from PHHs and HepG2 (Fig. 1C,D). HLCs and PHHs showed remarkable similarity in the levels of unsaturated residues, 14:1, 19:1, 20:2, 24:5 and 24:6 especially. A detailed analysis of the three different HLC lines, each obtained with a different differentiation method, showed how, for abundant residues, the residue content of HLCs was remarkably similar (Fig. 1B). Whereas, HLCs profiles for low abundance residues showed more differences, where 13:1, 15:0 and 20:0 average levels in HCL-5 were closer to PHHs than other HLCs. PCA analysis of residues was able to better highlight the differences among the three cell types (Fig. 1E). As confirmed by the residue analysis, differentiated hepatocytes showed the tendency to cluster among themselves, sharing features with both HepG2 and PHHs. This is less pronounced when the principal components are computed with all molecular lipids (Fig. 1F). We hypothesize that this is due to the whitening of the data performed during pre-processing. This step is performed to compensate the overwhelming breadth of lipid amounts, but in turns quantification errors from very low abundance compounds can be amplified in the computation of principal components. With liputils, similar residues are extracted from both very high- and very low-abundance residues, thus lessening the quantification errors from low-abundance analytes.

Residues extracted from the lipidomic data of the culture media showed that HepG2 medium contained remarkably higher concentrations and diversity of both abundant and less abundant residues compared to the media of the HLCs and PHHs. HepG2 medium contained 10% fetal bovine serum (FBS) comprised of unknown factors including complex lipids detectable by the mass spectrometry method used in the original study^31^. liputils showed that FAs 18:1, 16:0, 18:2 and 20:4 were the most abundant residues in the HepG2 medium (Supplementary Fig. S2) nicely in line with the FA content of the FBS reported elsewhere^32,33^. The media of the HLCs and PHHs were chemically defined and without animal serum explaining the difference observed in their level and diversity of detected residues compared to the HepG2 medium.

**Figure 2:**
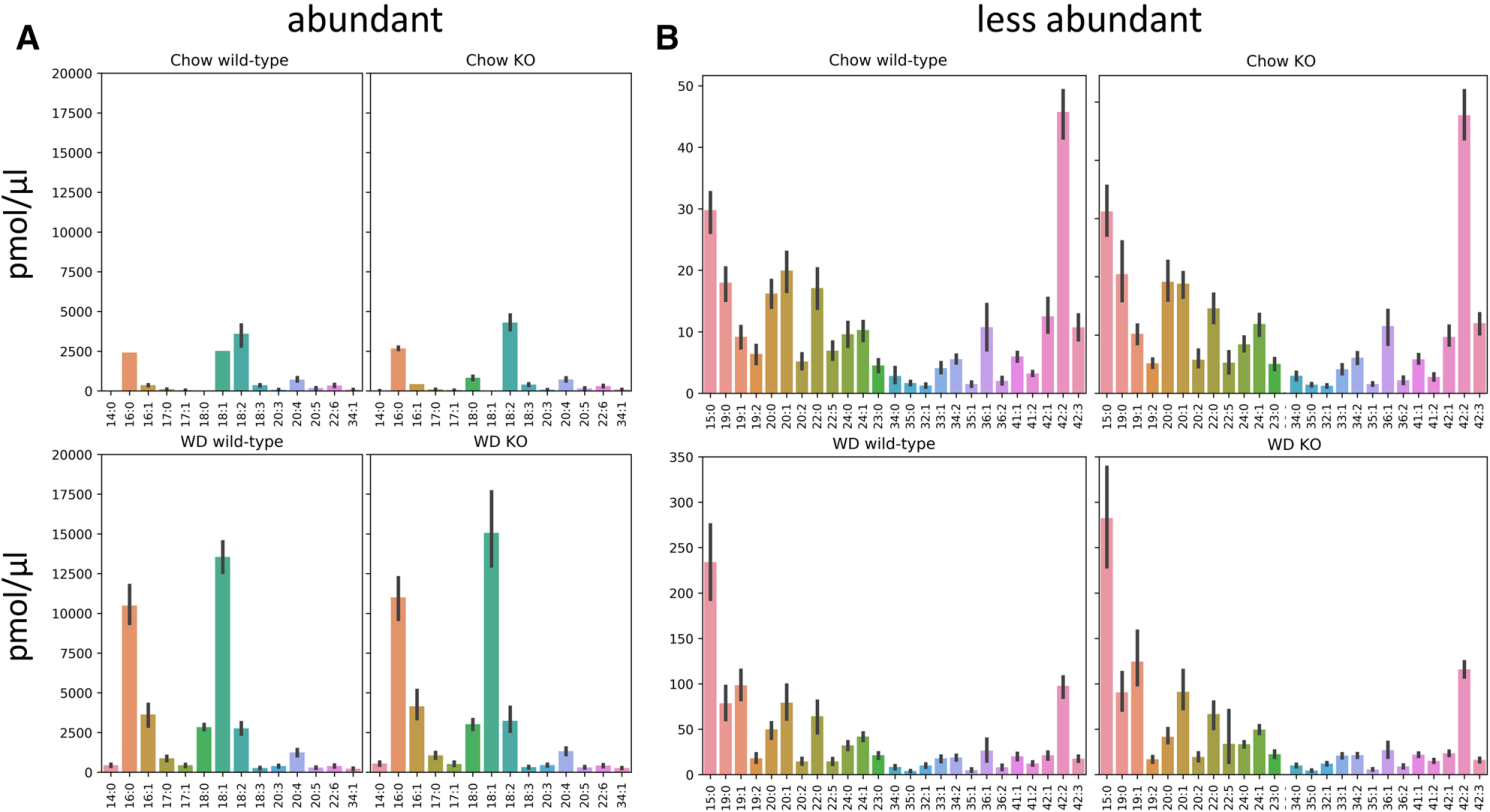
Lipidomics data obtained from plasma of animal models fed different diets is reprocessed with liputils. Residues extracted from liver lipidomics of Plpp3^+/+^ (wild-type) and Plpp3^−/−^ (KO), fed either Chow (top) or Western-type diet (WD, bottom) are shown (n = 9 mice per group). Abundant and less abundant residues are charted in (**A**) and (**B**), respectively. Units were unchanged during the extraction and are the same as the input data (pmol/ μl). Histograms show the average ± SD. There were no statistically significant differences among residues, diet-wise.

We next re-assessed lipidomic data from a finely-tuned in vivo gene editing experiment by our group^28^, where a specific lipid phosphate phosphatase, LPP3, was removed from the liver of adult mice by Cre-recombinase. Mice were then fed with either Chow or Western-type (WD) diet. Therein, we could pinpoint the activity of LPP3 to precise changes in the abundance of specific molecular lipids measured in the plasma, but in this case we would not expect that the ablation of hepatic LPP3—which acts on specific phosphorylated lipid substrates—had any impact on residue abundance. To this aim, we reprocessed the data with liputils. Data included LPP3 wild-type and LPP3 liver-specific deficient mice (the two genotypes of the study), diet-wise, for both abundant and rare lipid residues (Fig. 2A,B). Interestingly, we uncovered how the distribution of residues in the plasma was largely independent from the genotype. One notable exception was the artefactual residue 42:2, which was among the low-abundance residues but was higher at Chow than WD. This residue is reported by liputils from unresolved sphingomyelins mass isobars in the original lipidomic data, notably SM 42:2. This is not a limitation of liputils, but rather one of the resolution power of the original analysis.

**Figure 3:**
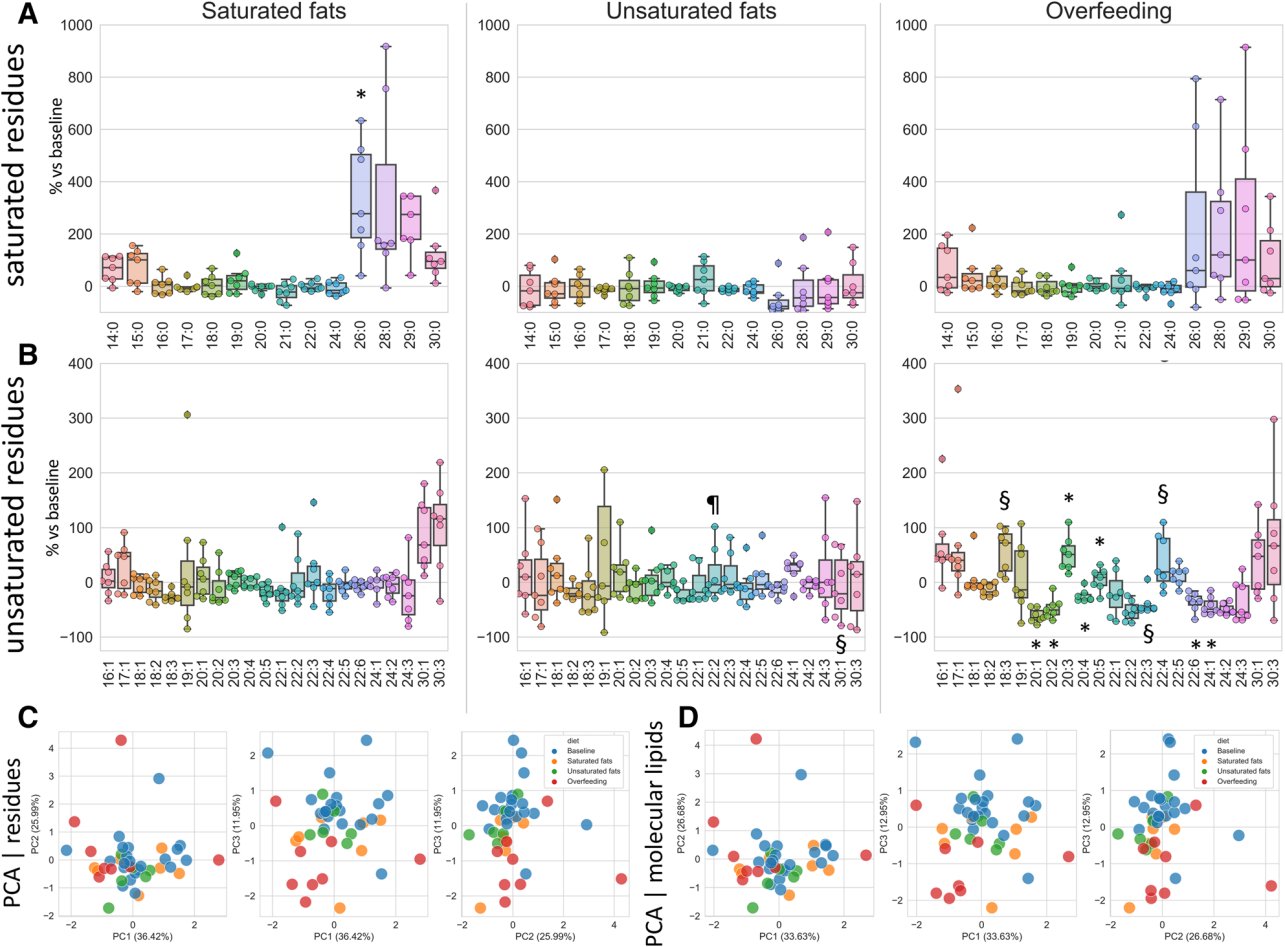
Fatty acid moieties percent changes among individuals following different dietary protocols with respect to baseline. Percentage changes of each dietary treatment (n = 7 subjects per group) with respect to its own baseline value is shown for each saturated (**A**) and unsaturated residue (**B**). (**A**) *p = 0.018 vs Unsaturated fats. (**B**) ^#§^p < 0.05 vs Saturated fats; *p < 0.05 vs Saturated fats and Unsaturated fats; ^¶^p = 0.017 vs Overfeeding. Statistically significant differences were determined with ANOVA followed by Tukey’s post-hoc test. Detailed statistics can be found in Supplementary Table S2. The upper and lower ends of the boxes indicate the 25th and 75th percentiles, respectively. The length of the box shows the interquartile range within which 50% of the values are located. The solid grey lines denote the median. Scatter plots of the first three principal components (PCs) are shown for residues of each sample (**C**) and for molecular lipids for each sample (**D**).

**Figure 4:**
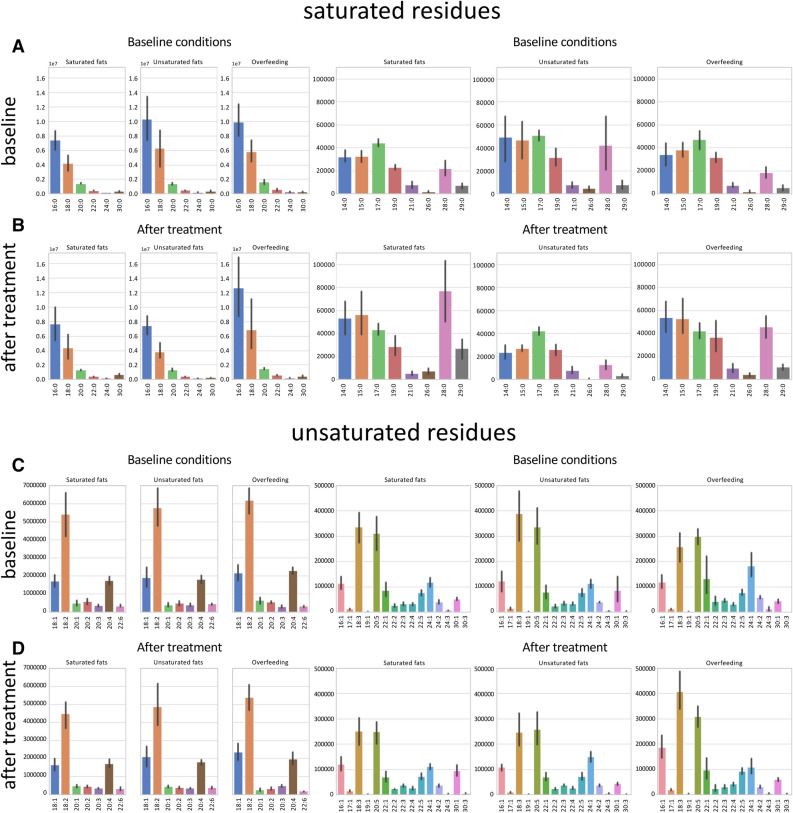
Distribution of fatty acid moieties among individuals at baseline and after different dietary protocols. Residues extracted from lipidomic data of human plasma (n = 7 subjects per group) are shown. Residues were divided into saturated residues (top, **A**,**B**) and unsaturated residues (bottom, **C**,**D**). There are three experimental conditions total: isocaloric diet with ~ 42.5% saturated fats (Saturated fats), ~ 42.5% monounsaturated fats (Unsaturated Fats) or 2000 kcal above baseline (Overfeeding). Each experimental condition has its own baseline. Histograms show the average ± SD.

A rich cache of lipidomic studies is hosted on Metabolomics Workbench (https://www.metabolomicsworkbench.org/). As a proof of principle of how liputils can be used to process lipidomic data that can be retrieved from there, we performed a full analysis of one published dataset (study ID ST000668, https://doi.org/10.21228/M85P6M; step-by-step instructions for the whole analysis are provided as a Jupyter Notebook in the Supplemental methods). Plasma from human subjects was subjected to lipidomics analysis at baseline (that is, prior to dietetic treatment), and after three different dietary intervention: either isocaloric diet with ~ 42.5% saturated fats (Saturated fats), ~ 42.5% monounsaturated fats (Unsaturated Fats) or 2000Kcal above baseline diet (Overfeeding). We included in our analysis all residues up 30 carbon atoms in length. This was done because the original data includes low-resolution compounds such as PE(P-40:6)/PE(O-40:7), that can be originating from a broad variety of compounds (Supplementary Fig. S3). According to data, the longest named fatty acids that could be distinguished without ambiguities were C-24 compounds, such as lignoceric acid and tetracosaenoic unsaturated members of the same length, so we chose C-30 as reasonable cutoff. Overall, no dietary treatment resulted in dramatic changes in the residue makeup (Figs. 3 and 4). Saturated fats diet, as well as Overfeeding, seemed to have an effect of boosting long-chain saturated residues (with respect to each subject’s baseline), but owing to small numerosity and variability, the trend was statistically significant for 26:0 only (Fig. 3A). The switch to either Saturated or Unsaturated fats diet had negligible results on plasma residue content (Fig. 3B). Conversely, Overfeeding had a considerable impact on residues longer than 20 carbon atoms (Fig. 3B), even if with mixed results. We hypothesize that this is mostly due to the very low levels of these residues (Fig. 4C,D), more prone to fluctuations and possibly upstream identification errors. A PCA performed on both residues and individual molecular lipids of the original data (Fig. 3C,D) is also suggestive that Overfeeding can have major impacts on plasma lipids than isocaloric saturated or unsaturated fatty acids containing diets.

In conclusion, we developed liputils, a Python library to ease the downstream analysis of individual fatty acid residues, in parallel and on top of completed lipidomic pipelines. liputils is primarily designed to work with RefMet-compliant nomenclature, even if it can process other types of annotations, including some used in commercial lipidomic services. Further, it is open source and actively maintained. We demonstrated how liputils could be used to get insights and explore lipidomic data from a different perspective and provided detailed protocols and examples with real world use cases.

## Supplementary information

Supplementary information 1

Supplementary information 2
