# Supplementary material for: liputils: a Python module to manage individual fatty acid moieties from complex lipids
Source: Sci Rep. 2020 Aug 7;10:13368. doi: 10.1038/s41598-020-70259-9 (PMC7415148; doi:10.1038/s41598-020-70259-9)

Example analysis of a publicly available dataset

This is an exploratory analysis of publicly available data from: [https://www.metabolomicsworkbench.org/data/DRCCMetadata.php?Mode=Study&StudyID=ST000668&StudyType=MS&ResultType=1 (https://www.metabolomicsworkbench.org/data/DRCCMetadata.php?Mode=Study&StudyID=ST000668&StudyType=MS&ResultType=1)](https://www.metabolomicsworkbench.org/data/DRCCMetadata.php?Mode=Study&StudyID=ST000668&StudyType=MS&ResultType=1)

As requested, we do include this statement that comes with data:

> This data is available at the NIH Common Fund's National Metabolomics Data Repository (NMDR) website, the Metabolomics Workbench, https://www.metabolomicsworkbench.org (https://www.metabolomicsworkbench.org), where it has been assigned Project ID PR000471. The data can be accessed directly via it's Project DOI: 10.21228/M85P6M This work is supported by NIH grant, U2C- DK119886.

Data preprocessing

In [1]:

```
# Author: stefano.manzini@gmail.com
# http://www.stemanz.altervista.org/
from matplotlib import pyplot as plt
%matplotlib inline
import seaborn as sns
from liputils import Lipid, make_residues_table
import pandas as pd
import numpy as np
from collections import Counter
import requests
```

Downloading the files from the repository in the local folder.

In [2]:

```
# data comes in two tables, from positive and negative ion modes

url1 = "https://www.metabolomicsworkbench.org/data/study_textformat_view.php?STU
url2 = "https://www.metabolomicsworkbench.org/data/study_textformat_view.php?STU

req1 = requests.get(url1, allow_redirects=False)
with open("ST000668_AN001022.csv", "wb") as o:
    o.write(req1.content)

req2 = requests.get(url2, allow_redirects=False)
with open("ST000668_AN001023.csv", "wb") as o:
    o.write(req2.content)
```

In [3]:

```
# manual inspection of the downloaded files is required to know how many rows
# need to be skipped to yield a non-malformed table
df1 = pd.read_csv("ST000668_AN001022.csv", sep="\t", skiprows=109)
df2 = pd.read_csv("ST000668_AN001023.csv", sep="\t", skiprows=109)
```

Even if it is hosted on Metabolomics Workbench, the is not 100% fully RefMet compliant. Before we use `liputils` , we want to use the online translator to convert all names into RefMet compliant ones. Before we do that, we must help the online translator by removing all unnecessary clutter from the analyte names.

In [4]:

```
# analytes contain information that prevent Metabolomics Workbench
# name translator from understanding who they are
# https://www.metabolomicsworkbench.org/databases/refmet/name_to_refmet.php
# example: CL 70:1; [M-2H](2-)@6.86
# it needs to be: CL 70:1

df1["Samples"] = df1["Samples"].apply(lambda x: x[:x.find(";")] if ";" in x else
df1["Samples"] = df1["Samples"].apply(lambda x: x[:x.find("[")] if "]" in x else
df2["Samples"] = df2["Samples"].apply(lambda x: x[:x.find(";")] if ";" in x else
df2["Samples"] = df2["Samples"].apply(lambda x: x[:x.find("[")] if "[" in x else
```

In [5]:

```
# the last two rows do not contain lipidomics data:
df1["Samples"][-3:]
```

Out[5]:

```
248    plasmenyl-PE 42:5
249      METABOLITES_END
250                 #END
Name: Samples, dtype: object
```

In [6]:

```
df1 = df1.iloc[:-2,:]
df2 = df2.iloc[:-2,:]
```

In [7]:

```
#checking if columns are equal. If they are, we merge the two dataframes
for x,y in zip(df1.columns, df2.columns):
    assert x == y
```

In [8]:

```
df = pd.concat([df1, df2], axis=0)
```

In [9]:

```
# this study uses a notation that is not RefMet compliant. We need to get
# online and retrieve translated terms from
# https://www.metabolomicsworkbench.org/databases/refmet/name_to_refmet.php
```

In [10]:

```
df = df.set_index("Samples")
```

In [11]:

```
# making a textfile with all analytes
with open("analytes.txt", "w") as o:
    for analyte in df.index:
        o.write(analyte+"\n")
```

In [12]:

```
# after manually inputting the analyte list in Metabolomics Workbench, a
# txt table of translated inputs is dowloaded. We load this table:
translated = pd.read_csv("refmet_results.txt", sep="\t")
translated.head(10)
```

Out[12]:

	Input name	RefMet name	Formula	Mass	REGNO
**0**	Factors	NaN	NaN	NaN	NaN
**1**	CL 70:1	CL(70:1)	C79H152O17P2	1435.0505	NaN
**2**	CL 70:5	CL(70:5)	C79H144O17P2	1426.9879	NaN
**3**	CL 74:1	CL(74:1)	C83H160O17P2	1491.1131	NaN
**4**	CL 74:7	CL(74:7)	C83H148O17P2	1479.0192	NaN
**5**	CL 82:13	NaN	NaN	NaN	NaN
**6**	FFA(20:0)	Arachidic acid	C20H40O2	312.3028	41.0
**7**	FFA(20:1)	Heneicosenoic acid	C21H40O2	324.3028	NaN
**8**	FFA(20:2)	Eicosadienoic acid	C20H36O2	308.2715	NaN
**9**	FFA(22:0)	Behenic acid	C22H44O2	340.3341	43.0

In [13]:

```
# not all inputs were translated (Factors, on row 0, is an artifact from the ol
translated.iloc[0,1] = "Factors" # replacing NaN with "Factors"
```

In [14]:

```
assert df.shape[0] == translated.shape[0]
```

In [15]:

```
# The tables have the same number of rows, so we can put the translated
# indices back into the former table, then drop all non translated
# compound (this is why we put back "Factors", as we still need to give labels
# to samples)
```

In [16]:

```
df["RefMet name"] = list(translated["RefMet name"])
df = df.dropna()
print(f"Total number of lipids: {df.shape[0]}")
```

```
Total number of lipids: 482
```

In [17]:

```
# it would be a good thing to rapidly identify each sample in terms of treatmen
# as well as stratify data. Let's get to know the different experimental condit
print(f"These are the possible experimental conditions:\n{'='*80}")
for c in set(df.loc["Factors",df.columns[:-1]]):
    print(c)
```

```
These are the possible experimental conditions:
======================================================================
==========
Diet intervention:Baseline | Gender:male
Diet intervention:Unsaturated Fatty Acid | Gender:female
Diet intervention:Saturated Fatty Acid | Gender:female
Diet intervention:Baseline | Gender:female
Diet intervention:Overfeeding-2 weeks | Gender:female
Diet intervention:Overfeeding-2 weeks | Gender:male
```

In [18]:

```
# we are going to modify the table a bit, so we save some info about
# the samples for future reference
sample_book = {k:v for k,v in zip(df.columns[:-1], df.loc["Factors", df.columns
```

In [19]:

```
# final preparation of the table for residue extraction. Setting the index with
# RefMet compliant lipid names
df = df.set_index("RefMet name")
```

In [20]:

```
df = df.drop(["Factors"])
```

Please note!

We need to **make sure that numeric data gets interpreted as numeric**, otherwise it will be discarded later on.

In [21]:

```
# liputils drops any non-numerical column by default.
# pandas has not interpreted the numbers in the table as numbers,
# so before proceeding we tell it that the table actually contains numbers.
df = df.astype("float64")
```

In [22]:

```python
# This is what the final, polished table looks like
df.head()
```

Out[22]:

RefMet name	S00019182	S00019183	S00019184	S00019185	S00019186	S00019187
**CL(70:1)**	1.415543e+05	1.356717e+05	4.174906e+04	4.683934e+04	6.963792e+04	5.236800e+04
**CL(70:5)**	5.700773e+04	2.957225e+04	1.253442e+04	9.635603e+03	2.957225e+04	1.524695e+04
**CL(74:1)**	4.295867e+04	5.400946e+04	2.451510e+04	3.276228e+04	4.427461e+04	2.548709e+04
**CL(74:7)**	6.722344e+04	1.681784e+04	5.551705e+04	2.618583e+04	4.973006e+04	2.618583e+04
**Arachidic acid**	1.701166e+06	1.161941e+06	1.399456e+06	1.399456e+06	1.399456e+06	1.399456e+06

5 rows × 42 columns

Extraction of lipid residues with liputils

We directly feed our data table to `make_residues_table()`. This function returns a `pandas.DataFrame` and expects analytes as row index and samples as column index.

In [23]:

```python
res = make_residues_table(df)

# residues are pulled in appearance order, so they need to be ordered
res = res.sort_index()
res.head()
```

Out[23]:

	S00019182	S00019183	S00019184	S00019185	S00019186	S00019187	S[illegible]
**14:0**	3.474830e+04	4.356682e+04	2.608958e+04	3.404043e+04	2.561295e+04	2.388370e+04	2.7[illegible]
**15:0**	2.600530e+04	2.938390e+04	4.065718e+04	4.502925e+04	2.922490e+04	2.326481e+04	2.2[illegible]
**16:0**	7.674743e+06	5.166182e+06	9.504088e+06	7.029468e+06	5.438244e+06	3.908616e+06	4.4[illegible]
**16:1**	7.453597e+04	9.748588e+04	1.343240e+05	1.057079e+05	1.047244e+05	7.011630e+04	8.6[illegible]
**17:0**	5.063393e+04	4.928738e+04	4.719015e+04	4.265817e+04	4.911188e+04	3.892206e+04	3.9[illegible]

5 rows × 42 columns

In [24]:

```
print(f"A total of {len(res.index)} individual residues have been extracted.")
```

```
A total of 212 individual residues have been extracted.
```

In [25]:

```
# switching rows to columns for easier plotting
data = res.T

# giving some labels
def get_diet(stringlike):
    if "Overfeeding-2 weeks" in stringlike:
        return "Overfeeding"
    elif "Baseline" in stringlike:
        return "Baseline"
    elif "Unsaturated" in stringlike:
        return "Unsaturated fats"
    elif "Saturated" in stringlike:
        return"Saturated fats"
    else:
        return "unrecognized diet"


# adding some labels to data
data["sex"]  = ["female" if "female" in sample_book.get(x) else "male" for x in
data["diet"] = [get_diet(sample_book.get(x)) for x in data.index]
```

PCA | residues

In [26]:

```
from sklearn.decomposition import PCA

pca = PCA(3, whiten=True)

pc = pca.fit_transform(res.T)

repack = pd.DataFrame(pc, columns=["PC1", "PC2", "PC3"])
repack["samples"] = list(res.T.index)
repack["diet"] = [get_diet(sample_book.get(x)) for x in res.T.index]
repack["sex"] = ["female" if "female" in sample_book.get(x) else "male" for x in
repack = repack.set_index("samples")
```

In [27]:

```python
perc1, perc2, perc3 = pca.explained_variance_ratio_
expl_var = {"PC1": perc1,
            "PC2": perc2,
            "PC3": perc3,
           }

# figure
fig, axes = plt.subplots(1, 3, figsize=(15,5))

for i, coord in enumerate((("PC1","PC2"),("PC1", "PC3"),("PC2", "PC3"))):
    x, y = coord
    axes[i].set_title(f"Principal components: {x}, {y}")

    scattter = sns.scatterplot(
        x=x, y=y, hue="diet", data=repack, s=300, ax=axes[i], alpha=.75
    )

    axes[i].set_xlabel(f"{x} ({round(expl_var.get(x)*100, 2)}%)", size=14)
    axes[i].tick_params(axis='both', which='major', labelsize=14)
    axes[i].set_ylabel(f"{y} ({round(expl_var.get(y)*100, 2)}%)", size=14)
    axes[i].legend().set_visible(False)

    fig.suptitle("PCA | Residues", size=20, y=1)
```

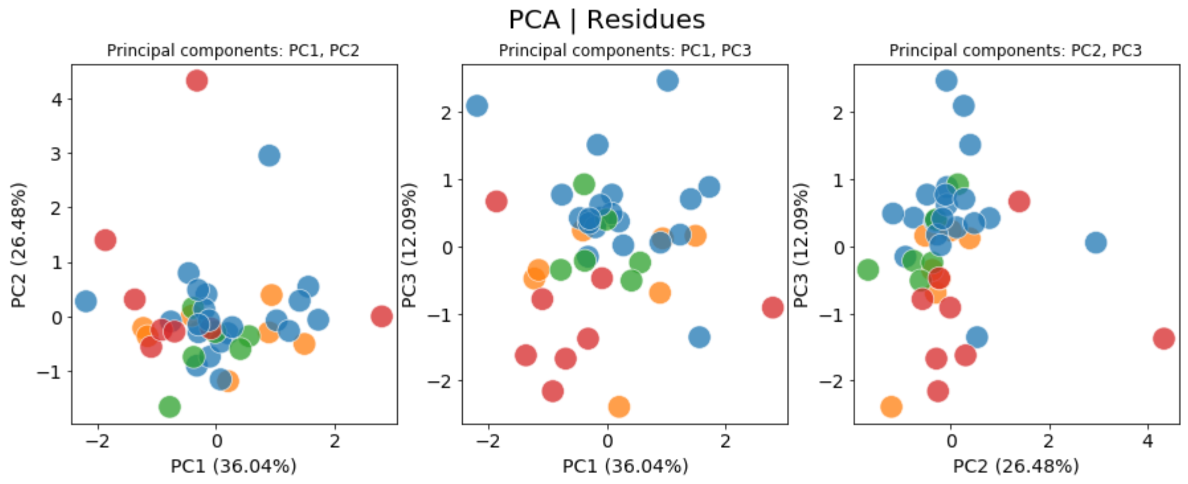

In [28]:

```python
from mpl_toolkits.mplot3d import Axes3D

sns.set_style("whitegrid")

repacked = repack.copy() # saves time, code reuse
if True:
    fig = plt.figure(figsize=(10,10))
    ax = fig.add_subplot(111, projection='3d')

    for g in set(repacked["diet"]):
        #c = colors.get(g)
        #m = marker.get(g)
        #print(f"Group: {g}, color: {c}")
        ax.scatter(
            repacked[repacked["diet"] == g]["PC1"], # x
            repacked[repacked["diet"] == g]["PC2"], # y
            repacked[repacked["diet"] == g]["PC3"], # z
            #c=c, #color
            s=550, #size
            alpha=.85,
            #marker = m,
        )

    #ax.view_init(25, 33) #azim=35
    ax.view_init(45, 45) #azim=35
print(f"Explained variance ratio: PC1: {round(perc1, 2)*100}%, PC2: {round(perc[illegible]
```

```
Explained variance ratio: PC1: 36.0%, PC2: 26.0%, PC3: 36.0%,
```

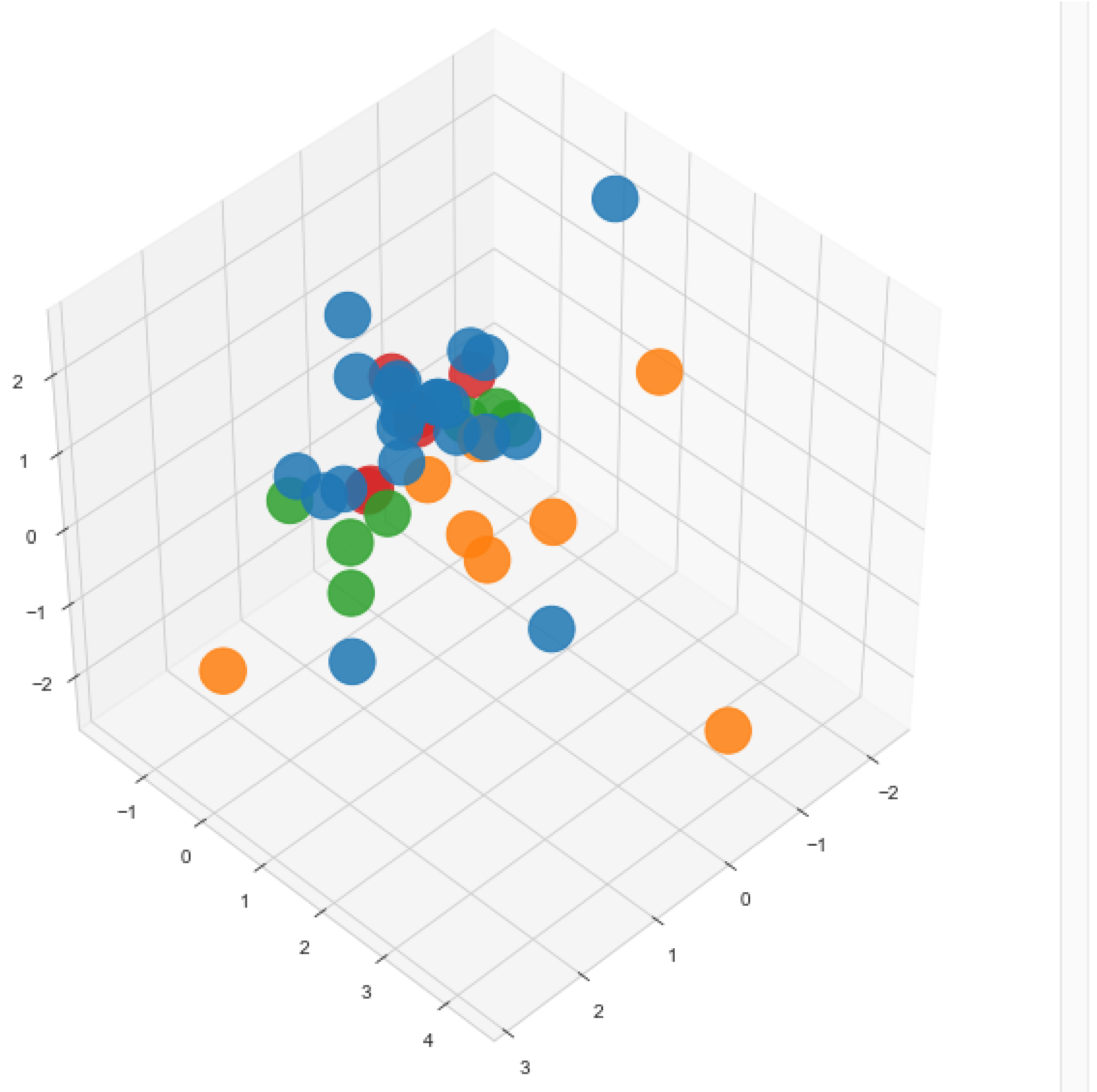

PCA | molecular lipids

In [29]:

```
pca = PCA(3, whiten=True)

pc = pca.fit_transform(df.T)

repack = pd.DataFrame(pc, columns=["PC1", "PC2", "PC3"])
repack["samples"] = list(df.T.index)
repack["diet"] = [get_diet(sample_book.get(x)) for x in df.T.index]
repack["sex"] = ["female" if "female" in sample_book.get(x) else "male" for x in
repack = repack.set_index("samples")
```

In [30]:

```
perc1, perc2, perc3 = pca.explained_variance_ratio_
expl_var = {"PC1": perc1,
            "PC2": perc2,
            "PC3": perc3,
           }

# figure
fig, axes = plt.subplots(1, 3, figsize=(15,5))

for i, coord in enumerate((("PC1","PC2"),("PC1", "PC3"),("PC2", "PC3"))):
    x, y = coord
    axes[i].set_title(f"Principal components: {x}, {y}")

    scattter = sns.scatterplot(
        x=x, y=y, hue="diet", data=repack, s=300, ax=axes[i], alpha=.75
    )

    axes[i].set_xlabel(f"{x} ({round(expl_var.get(x)*100, 2)}%)", size=14)
    axes[i].tick_params(axis='both', which='major', labelsize=14)
    axes[i].set_ylabel(f"{y} ({round(expl_var.get(y)*100, 2)}%)", size=14)
    axes[i].legend().set_visible(False)

    fig.suptitle("PCA | Molecular lipids", size=20, y=1)
```

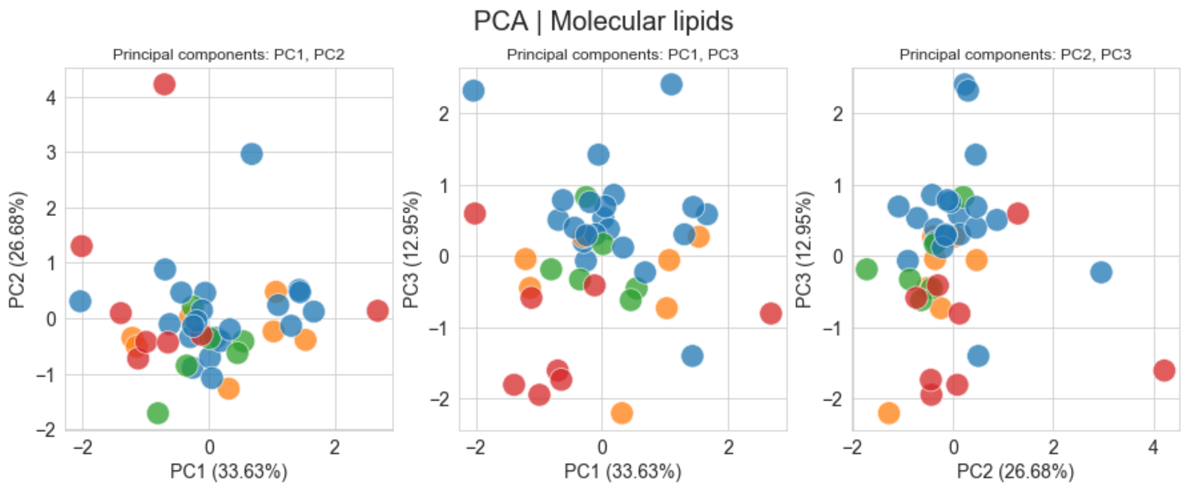

In [31]:

```python
from mpl_toolkits.mplot3d import Axes3D

sns.set_style("whitegrid")


repacked = repack.copy() # saves time, code reuse
if True:
    fig = plt.figure(figsize=(10,10))
    ax = fig.add_subplot(111, projection='3d')

    for g in set(repacked["diet"]):
        #c = colors.get(g)
        #m = marker.get(g)
        #print(f"Group: {g}, color: {c}")
        ax.scatter(
            repacked[repacked["diet"] == g]["PC1"], # x
            repacked[repacked["diet"] == g]["PC2"], # y
            repacked[repacked["diet"] == g]["PC3"], # z
            #c=c, #color
            s=550, #size
            alpha=.85,
            #marker = m,
        )
    # curioso plot dei punti nella griglia..

    #ax.view_init(25, 33) #azim=35
    ax.view_init(45, 45) #azim=35
print(f"Explained variance ratio: PC1: {round(perc1, 2)*100}%, PC2: {round(perc2
```

```
Explained variance ratio: PC1: 34.0%, PC2: 27.0%, PC3: 33.6%,
```

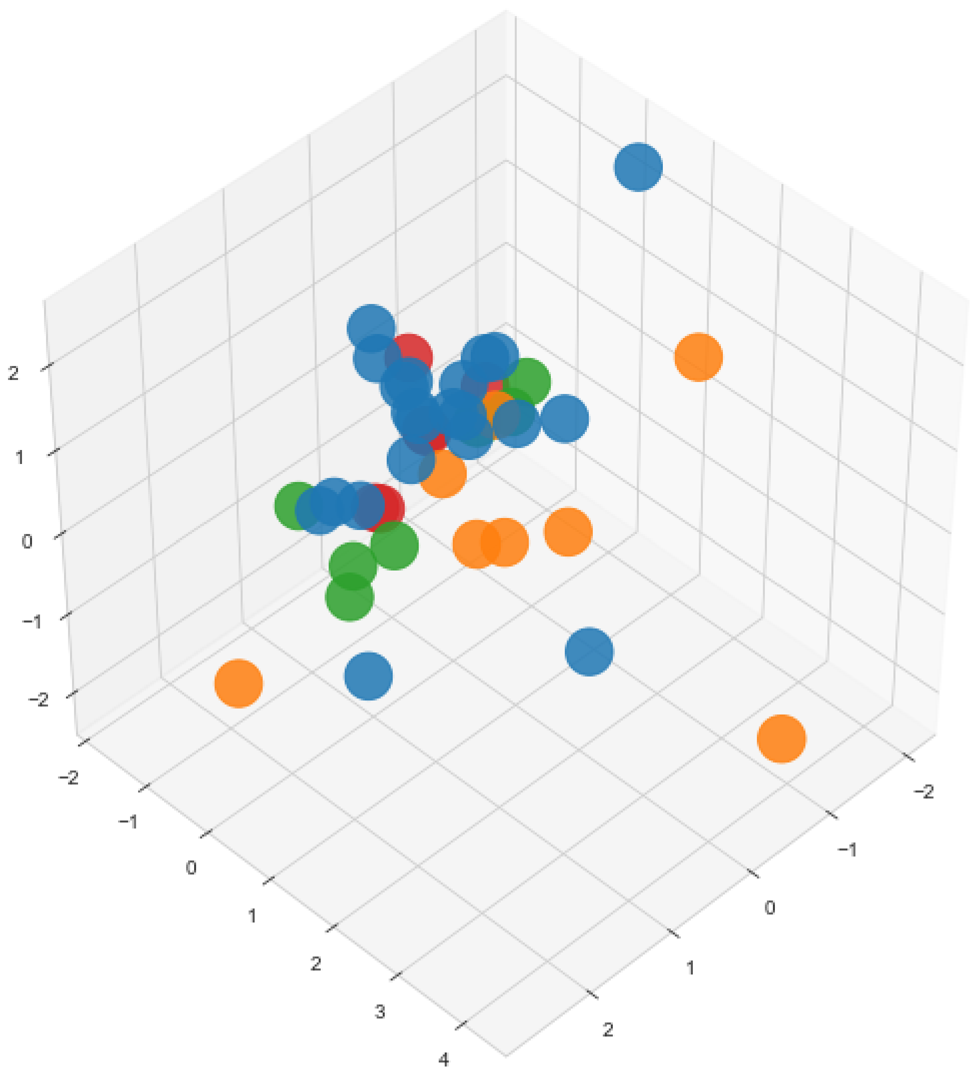

Per-sample percentage change over baseline

A little more pre-processing

Each subject has two measures: baseline and treatment. We need to pair them to further the analysis. The files that are downloaded from the study contain this information, but it is cumbersome to read it with `pandas` . Instead we pull it from the [Project's page (https://www.metabolomicsworkbench.org/data/subject_fetch.php?STUDY_ID=ST000668&STUDY_TYPE=MS&RESULT_TYPE=1)](https://www.metabolomicsworkbench.org/data/subject_fetch.php?STUDY_ID=ST000668&STUDY_TYPE=MS&RESULT_TYPE=1):

There's no direct handle so we copypaste it into a new spreadsheet.

In [32]:

```
subjects = pd.read_csv("samples and subjects.txt", sep="\t")
```

In [33]:

```
subjects.head(2)
```

Out[33]:

	mb_sample_id	Subject name	Sample name	Diet intervention	Gender
**0**	SA038455	SU0019532	S00019182	Baseline	female
**1**	SA038467	SU0019533	S00019184	Baseline	female

Each sample has its own unique ID, but let's check whether there are two measurement per sample:

In [34]:

```
c = Counter(subjects["Subject name"])
c # for each subject, we have two measures
```

Out[34]:

```
Counter({'SU0019532': 2,
         'SU0019533': 2,
         'SU0019534': 2,
         'SU0019535': 2,
         'SU0019536': 2,
         'SU0019537': 2,
         'SU0019538': 2,
         'SU0019539': 2,
         'SU0019540': 2,
         'SU0019541': 2,
         'SU0019542': 2,
         'SU0019543': 2,
         'SU0019544': 2,
         'SU0019545': 2,
         'SU0019548': 2,
         'SU0019549': 2,
         'SU0019553': 2,
         'SU0019546': 2,
         'SU0019547': 2,
         'SU0019550': 2,
         'SU0019551': 2,
         'SU0019552': 2})
```

We now need to process our data so that the columns that hold the samples are paired, then the percentage change is calculated.

In [35]:

```
subjects = subjects.set_index("Subject name")

subjects_book = {}
for k in c:
    subjects_book.setdefault(k, list(subjects.loc[k]["Sample name"]))
```

In [36]:

```
subjects_book
```

Out[36]:

```
{'SU0019532': ['S00019182', 'S00019183'],
 'SU0019533': ['S00019184', 'S00019185'],
 'SU0019534': ['S00019186', 'S00019187'],
 'SU0019535': ['S00019188', 'S00019189'],
 'SU0019536': ['S00019190', 'S00019191'],
 'SU0019537': ['S00019192', 'S00019193'],
 'SU0019538': ['S00019194', 'S00019195'],
 'SU0019539': ['S00019196', 'S00019197'],
 'SU0019540': ['S00019198', 'S00019199'],
 'SU0019541': ['S00019200', 'S00019201'],
 'SU0019542': ['S00019202', 'S00019203'],
 'SU0019543': ['S00019204', 'S00019205'],
 'SU0019544': ['S00019206', 'S00019207'],
 'SU0019545': ['S00019208', 'S00019209'],
 'SU0019548': ['S00019214', 'S00019215'],
 'SU0019549': ['S00019216', 'S00019217'],
 'SU0019553': ['S00019224', 'S00019225'],
 'SU0019546': ['S00019210', 'S00019211'],
 'SU0019547': ['S00019212', 'S00019213'],
 'SU0019550': ['S00019218', 'S00019219'],
 'SU0019551': ['S00019220', 'S00019221'],
 'SU0019552': ['S00019222', 'S00019223']}
```

Smartly enough, the baseline conditions are even numbered, and post-treatment values are odd numbered. So we can easily check above that the samples were correctly paired.

In [37]:

```
templist = []
for i, k in enumerate(subjects_book.keys()):
    try:
        col1, col2 = subjects_book.get(k)
        base, treatment = res[col1], res[col2]
        templist.append(((treatment - base)/base)*100)
    except KeyError:
        print(f"Warning: {k} can't be found among samples ?!")
print(f"Processed {i+1} entries")
```

```
Warning: SU0019540 can't be found among samples ?!
Processed 22 entries
```

One pair of samples is listed in the project's files, but it is not present in the project's data.

Final prepping of data before plotting:

In [38]:

```
residue_perc = pd.concat(templist, axis=1)
newcols = {old: new for old, new in zip(residue_perc.columns, subjects_book.keys

residue_perc = residue_perc.rename(columns=newcols)

dfperc = residue_perc.T
dfperc["diet"] = [list(subjects.loc[key]["Diet intervention"])[1] for key in dfp
```

In [39]:

```
paper_names = {
    "Saturated Fatty Acid" : "Saturated fats",
    "Unsaturated Fatty Acid" : "Unsaturated fats",
    "Overfeeding-2 weeks" : "Overfeeding",
}
```

In [40]:

```
dfperc["diet"] = dfperc["diet"].map(paper_names)
```

Percent change over baseline plots

We are using two `liputils` functions that help managing the residues that we want to plot:

`saturated()` returns `True` if a residue is saturated, and `False` otherwise. This is convenient as unsaturated residues can be speficied via the `not` operator, as in `not saturated(residue)`.

`max_carbon()` returns `True` is the specified residue is up the specified number of carbon atoms in lenght, `False` otherwise.

They can be combined to pythonically define the residue subset that needs plotting, like in here (line 22):

In [41]:

```
from liputils import saturated, max_carbon
```

In [42]:

```python
case = "saturated residues percentages"

fig, axes = plt.subplots(1, 3, figsize=(20,5))
label = "diet"
labelled_columns = 1
TITLE_SIZE = 22
TICKSIZE = 14

plt.suptitle(case, y=1.05, size=TITLE_SIZE*1.13)
for i, group in enumerate(('Saturated fats', 'Unsaturated fats', 'Overfeeding'))

    axes[i].set_title(f"{group}", size=TITLE_SIZE)

    # applying a threshold.
    # ==================================================
    cols = abs(dfperc._get_numeric_data().max()) >= 0 # that is, all of them
    cols_to_keep = cols[cols].index
    table = dfperc[cols_to_keep]

    # saturated or insaturated?
    # =========================
    table = table.loc[:,[saturated(x) and max_carbon(x, 30) for x in table.colum

    # putting back sample labels
    table = pd.concat([table, dfperc[dfperc.columns[-labelled_columns:]]], axis=

    table=table[table[label] == group]

    # we needed to put back the labels to drop non-group rows, now
    # we just need to plot the numbers
    table=table[table.columns[:-labelled_columns]] # only residue columns

    #bar = sns.barplot(data=table, ax=axes[i])
    box = sns.boxplot(data=table, ax=axes[i], whis=1.5)
    for patch in box.artists:
        r, g, b, a = patch.get_facecolor()
        patch.set_facecolor((r, g, b, .5))
    swarm = sns.swarmplot(data=table, ax=axes[i], s=6, alpha=.8, edgecolor="blac
    axes[i].tick_params(rotation=90, labelsize=TICKSIZE, axis="x")
    axes[i].tick_params(labelsize=TICKSIZE, axis="y")
    axes[i].set_ylim(-100, 1000)
```

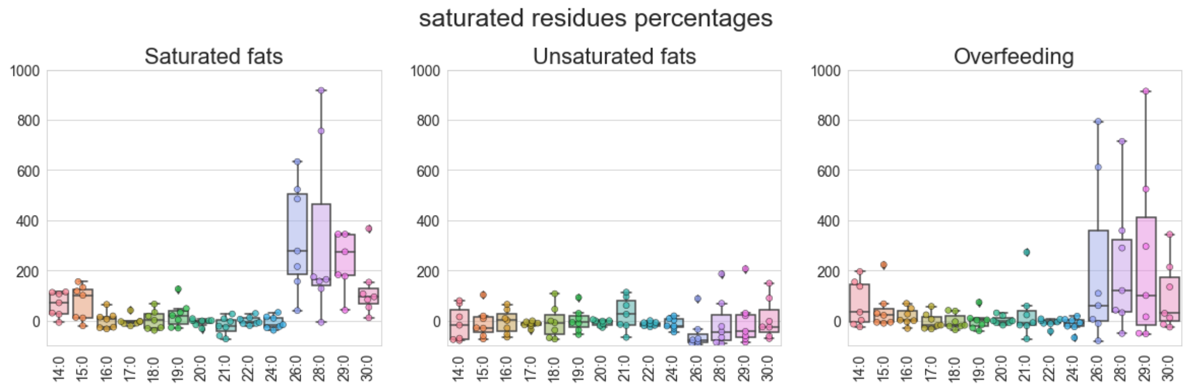

In [43]:

```python
case = "unsaturated residues percentages"

fig, axes = plt.subplots(1, 3, figsize=(20,5))
label = "diet"
labelled_columns = 1
TITLE_SIZE = 22
TICKSIZE = 14

plt.suptitle(case, y=1.05, size=TITLE_SIZE*1.13)
for i, group in enumerate(('Saturated fats', 'Unsaturated fats', 'Overfeeding'))

    axes[i].set_title(f"{group}")

    # applying a threshold.
    # ==================================================
    cols = abs(dfperc._get_numeric_data().max()) >= 0 # that is, all of them
    cols_to_keep = cols[cols].index
    table = dfperc[cols_to_keep]

    # saturated or insaturated?
    # =========================
    table = table.loc[:,[not saturated(x) and max_carbon(x, 30) for x in table.

    # putting back sample labels
    table = pd.concat([table, dfperc[dfperc.columns[-labelled_columns:]]], axis=

    table=table[table[label] == group]

    # we needed to put back the labels to drop non-group rows, now
    # we just need to plot the numbers
    table=table[table.columns[:-labelled_columns]] # only residue columns

    #bar = sns.barplot(data=table, ax=axes[i])
    box = sns.boxplot(data=table, ax=axes[i], whis=2)
    for patch in box.artists:
        r, g, b, a = patch.get_facecolor()
        patch.set_facecolor((r, g, b, .5))
    swarm = sns.swarmplot(data=table, ax=axes[i], s=6, alpha=.8, edgecolor="blac
    axes[i].tick_params(rotation=90, labelsize=TICKSIZE, axis="x")
    axes[i].tick_params(labelsize=TICKSIZE, axis="y")
    axes[i].set_ylim(-125, 1000)

print(case)
#plt.savefig(f"/Users/manz/Desktop/{case}.png", dpi=600, bbox_inches="tight")
```

```
unsaturated residues percentages
```

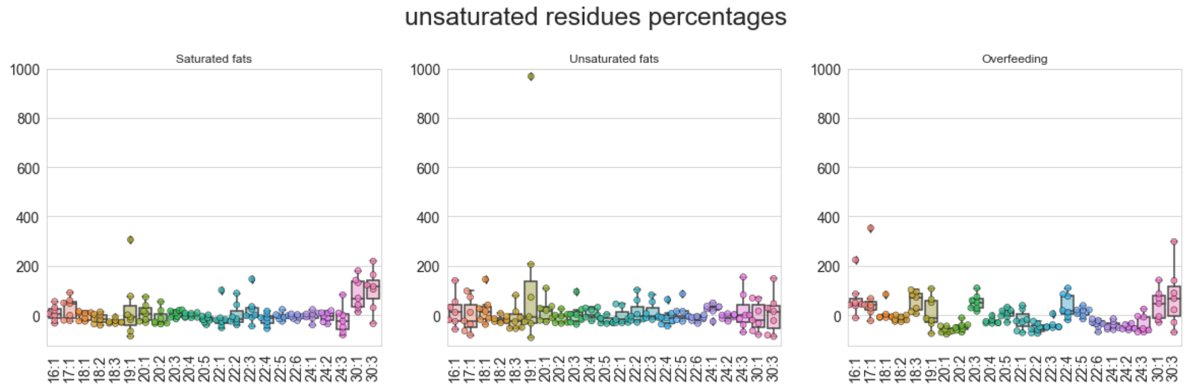

There's a clear outlier in the 19:1 residue in Unsaturated fats, that renders the figure hard to read, flattening all the boxes. Let's reduce the span of y-axis:

In [44]:

```
case = "unsaturated residues percentages (capped)"

fig, axes = plt.subplots(1, 3, figsize=(20,5))
label = "diet"
labelled_columns = 1
TITLE_SIZE = 22
TICKSIZE = 14

plt.suptitle(case, y=1.05, size=TITLE_SIZE*1.13)
for i, group in enumerate(('Saturated fats', 'Unsaturated fats', 'Overfeeding'))

    axes[i].set_title(f"{group}", size=TITLE_SIZE)

    # applying a threshold.
    # ==================================================
    cols = abs(dfperc._get_numeric_data().max()) >= 0 # that is, all of them
    cols_to_keep = cols[cols].index
    table = dfperc[cols_to_keep]

    # saturated or insaturated?
    # =========================
    table = table.loc[:,[not saturated(x) and max_carbon(x, 30) for x in table.

    # putting back sample labels
    table = pd.concat([table, dfperc[dfperc.columns[-labelled_columns:]]], axis=

    table=table[table[label] == group]

    # we needed to put back the labels to drop non-group rows, now
    # we just need to plot the numbers
    table=table[table.columns[:-labelled_columns]] # only residue columns

    #bar = sns.barplot(data=table, ax=axes[i])
    box = sns.boxplot(data=table, ax=axes[i], whis=2)
    for patch in box.artists:
        r, g, b, a = patch.get_facecolor()
        patch.set_facecolor((r, g, b, .5))
    swarm = sns.swarmplot(data=table, ax=axes[i], s=6, alpha=.8, edgecolor="blac
    axes[i].tick_params(rotation=90, labelsize=TICKSIZE, axis="x")
    axes[i].tick_params(labelsize=TICKSIZE, axis="y")
    axes[i].set_ylim(-125, 400)
```

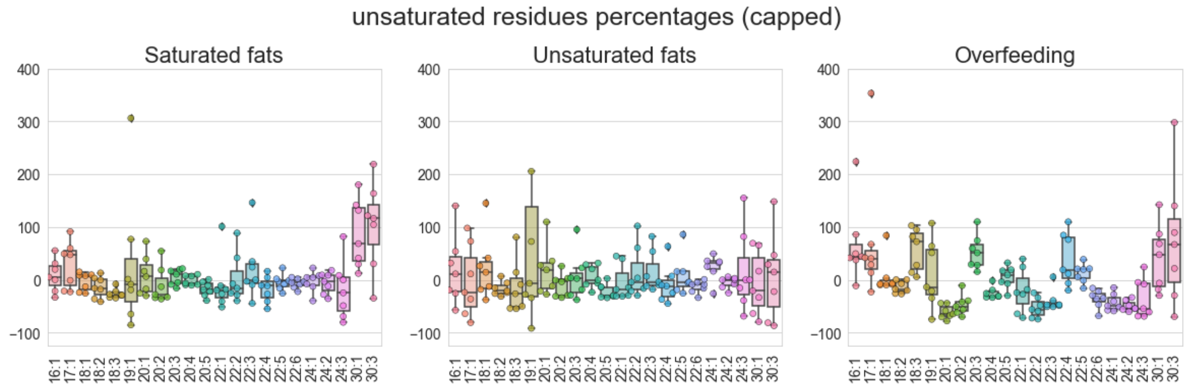

This plot look definitely better than the previous one.

Residues bar plots

Baseline and treatment

Residue abundance vary greatly. An arbitrary threshold has been chosen to differentiate "abundant" and "less abundant" residues. Charts thus have different y-axis maxima, just for the sake of clarity.

Plotting saturated residues

In [45]:

```
# taken from online sample details
baseline_samples = {
"Overfeeding" : ['S00019214', 'S00019216', 'S00019218', 'S00019220',
                 'S00019222', 'S00019224'],
"Unsaturated fats" : ['S00019196', 'S00019200', 'S00019202', 'S00019204',
                      'S00019206', 'S00019208',],
"Saturated fats" : ['S00019182', 'S00019184', 'S00019186', 'S00019188',
                    'S00019190', 'S00019192', 'S00019194'],
}
```

In [46]:

```
label = "Baseline conditions, abundant, saturated residues"

fig, axes = plt.subplots(1, 3, figsize=(14,4))
plt.suptitle(label, size=18, y=1.05)
labelled_columns = 2

for i, key in enumerate(("Saturated fats", "Unsaturated fats", "Overfeeding")):

    axes[i].set_title(f"{key}")

    table = data.loc[baseline_samples.get(key), :].copy()

    # saturated or insaturated?
    # =========================
    table = table.loc[:,[saturated(x) and max_carbon(x, 30) for x in table._get_

    cols = table.max() < 100_000
    cols = cols[cols].index
    table = table[cols]
    BASELINE_COLS = cols.copy()

    bar = sns.barplot(data=table, ax=axes[i],
                      #alpha=.5
                     )
    #swarm = sns.swarmplot(data=table, ax=axes[i], s=10, alpha=.85, edgecolor="
    axes[i].tick_params(rotation=90, labelsize=10, axis="x")
    axes[i].tick_params(labelsize=10, axis="y")
    axes[i].set_ylim(0, 110_000)
```

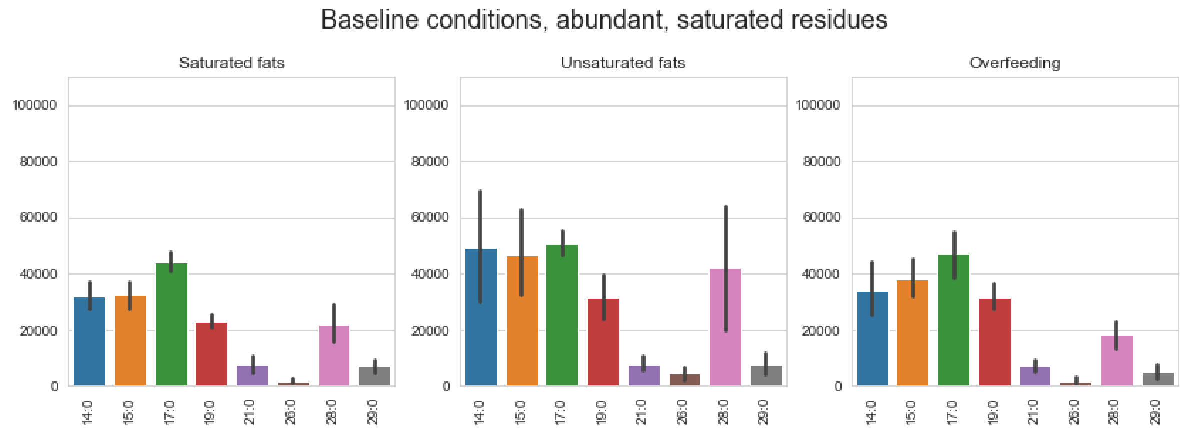

In [47]:

```python
label = "After treatment, abundant, saturated residues"

fig, axes = plt.subplots(1, 3, figsize=(14,4))
plt.suptitle(label, size=18, y=1.05)
labelled_columns = 2

for i, key in enumerate(("Saturated fats", "Unsaturated fats", "Overfeeding")):

    axes[i].set_title(f"{key}")

    #table = data.loc[baseline_samples.get(key), :].copy()
    table = data[data["diet"] == key].copy()

    # saturated or insaturated?
    # =========================
    table = table.loc[:,[saturated(x) and max_carbon(x, 30) for x in table._get_

    # the same as previous plot
    table = table[BASELINE_COLS]

    bar = sns.barplot(data=table, ax=axes[i],
                      #alpha=.5
                     )
    #swarm = sns.swarmplot(data=table, ax=axes[i], s=10, alpha=.85, edgecolor="
    axes[i].tick_params(rotation=90, labelsize=10, axis="x")
    axes[i].tick_params(labelsize=10, axis="y")
    axes[i].set_ylim(0, 110_000)
```

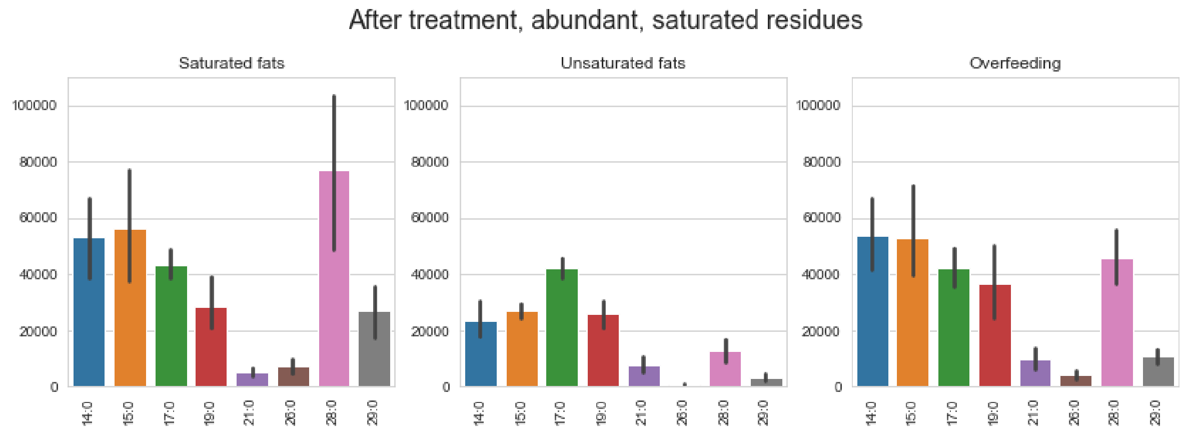

In [48]:

```
label = "Baseline conditions, less abundant, saturated residues"

fig, axes = plt.subplots(1, 3, figsize=(8,4))
plt.suptitle(label, size=18, y=1.05)
labelled_columns = 2

for i, key in enumerate(("Saturated fats", "Unsaturated fats", "Overfeeding")):

    axes[i].set_title(f"{key}", y=1.05)

    table = data.loc[baseline_samples.get(key), :].copy()

    # saturated or insaturated?
    # =========================
    table = table.loc[:,[saturated(x) and max_carbon(x, 30) for x in table._get_

    cols = table.max() >= 100_000
    cols = cols[cols].index
    table = table[cols]
    BASELINE_COLS = cols.copy()

    bar = sns.barplot(data=table, ax=axes[i],
                      #alpha=.5
                     )
    #swarm = sns.swarmplot(data=table, ax=axes[i], s=10, alpha=.85, edgecolor="
    axes[i].tick_params(rotation=90, labelsize=10, axis="x")
    axes[i].tick_params(labelsize=10, axis="y")
    axes[i].set_ylim(0, 17_500_000)
```

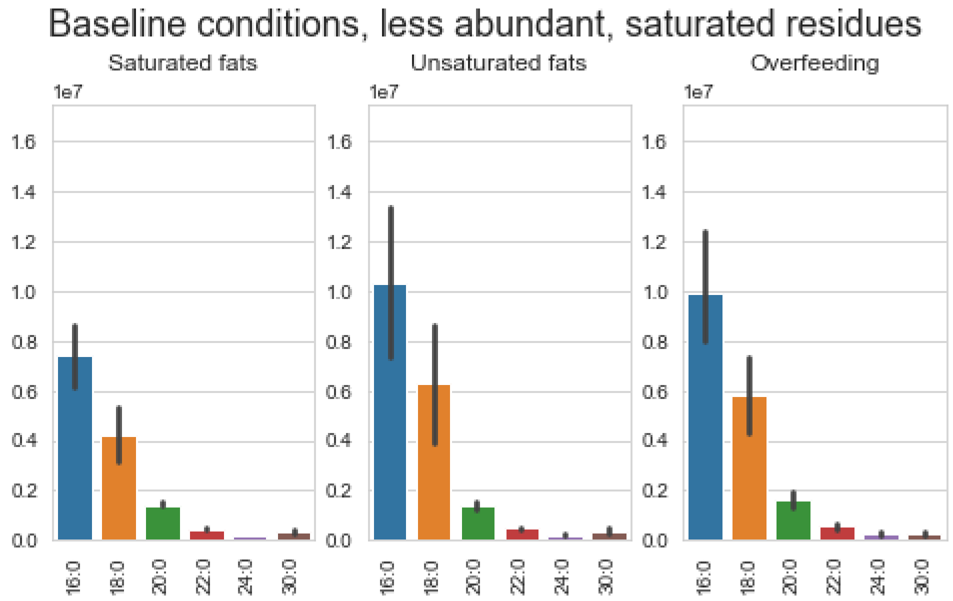

In [49]:

```
label = "After treatment, less abundant, saturated residues"

fig, axes = plt.subplots(1, 3, figsize=(8,4))
plt.suptitle(label, size=18, y=1.05)
labelled_columns = 2

for i, key in enumerate(("Saturated fats", "Unsaturated fats", "Overfeeding")):

    axes[i].set_title(f"{key}")

    #table = data.loc[baseline_samples.get(key), :].copy()
    table = data[data["diet"] == key].copy()

    # saturated or insaturated?
    # =========================
    table = table.loc[:,[saturated(x) and max_carbon(x, 30) for x in table._get_

    # the same as previous plot
    table = table[BASELINE_COLS]

    bar = sns.barplot(data=table, ax=axes[i],
                      #alpha=.5
                     )
    #swarm = sns.swarmplot(data=table, ax=axes[i], s=10, alpha=.85, edgecolor="
    axes[i].tick_params(rotation=90, labelsize=10, axis="x")
    axes[i].tick_params(labelsize=10, axis="y")
    axes[i].set_ylim(0, 17_500_000)
```

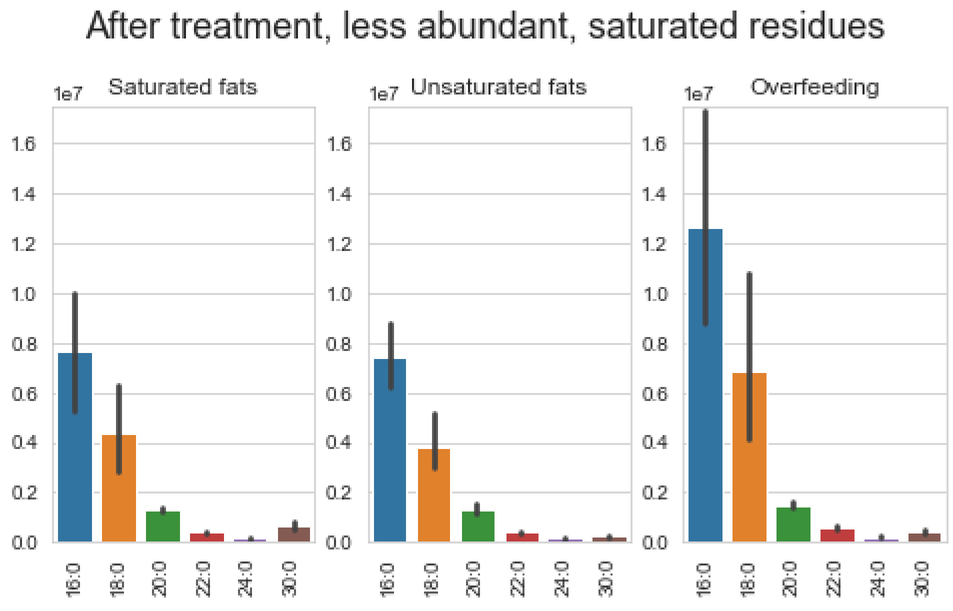

Plotting unsaturated residues

In [50]:

```python
label = "Baseline conditions, abundant, unsaturated residues"

fig, axes = plt.subplots(1, 3, figsize=(8,4))
plt.suptitle(label, size=18, y=1.05)
labelled_columns = 2

for i, key in enumerate(("Saturated fats", "Unsaturated fats", "Overfeeding")):

    axes[i].set_title(f"{key}")

    table = data.loc[baseline_samples.get(key), :].copy()

    # saturated or insaturated?
    # ==========================
    table = table.loc[:,[not saturated(x) and max_carbon(x, 30) for x in table._

    # these need to be kept the same manually
    if i == 0:
        cols = table.max() > 500_000
        cols = cols[cols].index
    table = table[cols]
    BASELINE_COLS = cols.copy()

    bar = sns.barplot(data=table, ax=axes[i],
                      #alpha=.5
                     )
    #swarm = sns.swarmplot(data=table, ax=axes[i], s=10, alpha=.85, edgecolor="
    axes[i].tick_params(rotation=90, labelsize=10, axis="x")
    axes[i].tick_params(labelsize=10, axis="y")
    if i != 0:
        axes[i].set_yticklabels([])
    axes[i].set_ylim(0, 7_000_000)
```

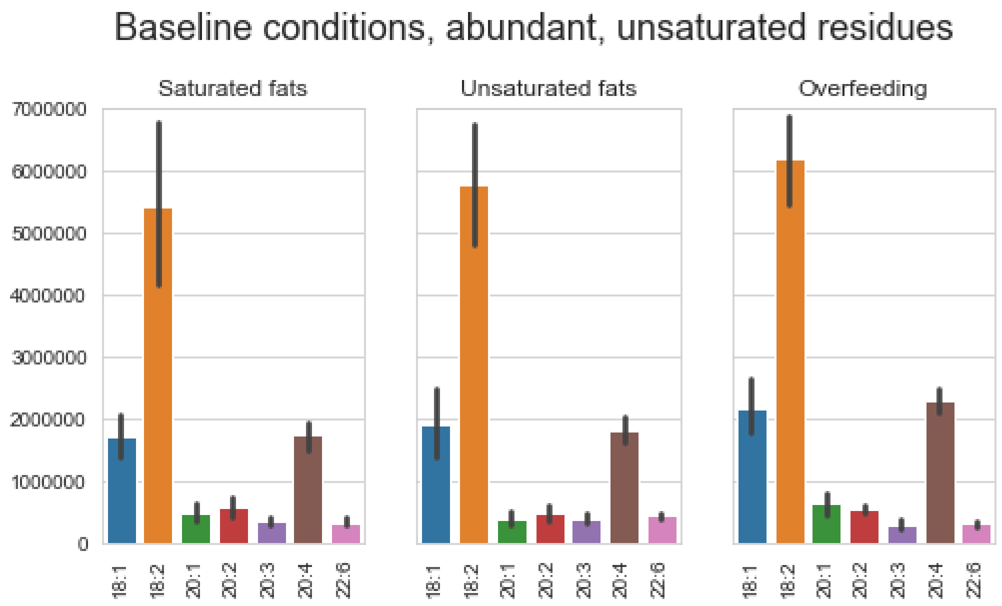

In [51]:

```
label = "After treatment, abundant, unsaturated residues"

fig, axes = plt.subplots(1, 3, figsize=(8,4))
plt.suptitle(label, size=18, y=1.05)
labelled_columns = 2

for i, key in enumerate(("Saturated fats", "Unsaturated fats", "Overfeeding")):

    axes[i].set_title(f"{key}")

    #table = data.loc[baseline_samples.get(key), :].copy()
    table = data[data["diet"] == key].copy()

    # the same as previous plot
    table = table[BASELINE_COLS]

    bar = sns.barplot(data=table, ax=axes[i],
                      #alpha=.5
                     )
    #swarm = sns.swarmplot(data=table, ax=axes[i], s=10, alpha=.85, edgecolor="
    axes[i].tick_params(rotation=90, labelsize=10, axis="x")
    axes[i].tick_params(labelsize=10, axis="y")
    if i != 0:
        axes[i].set_yticklabels([])
    axes[i].set_ylim(0, 7_000_000)
```

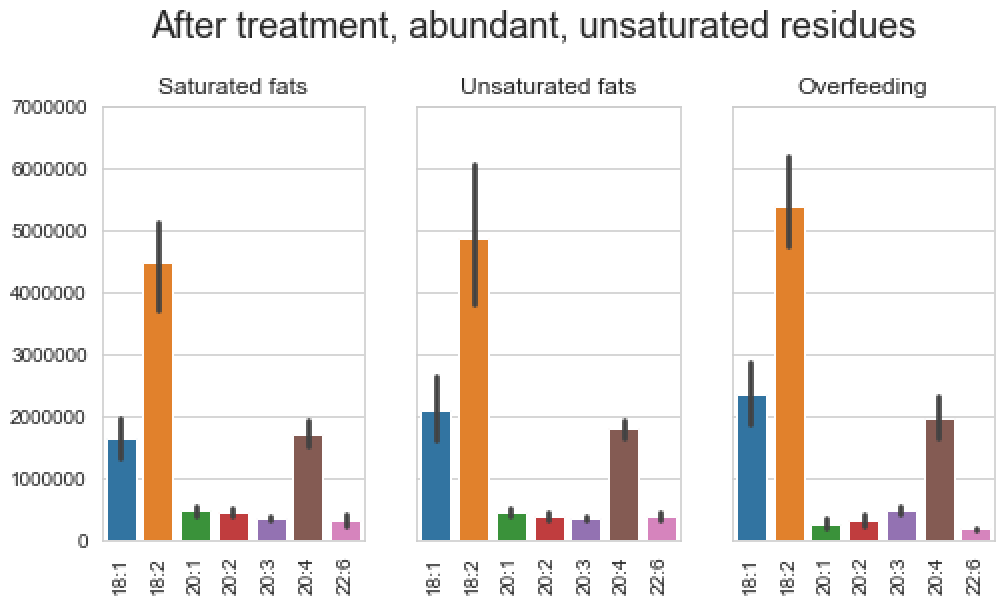

In [52]:

```python
label = "Baseline conditions, less abundant, unsaturated residues"

fig, axes = plt.subplots(1, 3, figsize=(14,4))
plt.suptitle(label, size=18, y=1.05)
labelled_columns = 2

for i, key in enumerate(("Saturated fats", "Unsaturated fats", "Overfeeding")):

    axes[i].set_title(f"{key}")

    table = data.loc[baseline_samples.get(key), :].copy()

    # saturated or insaturated?
    # =========================
    table = table.loc[:,[not saturated(x) and max_carbon(x, 30) for x in table._

    # these need to be kept the same manually
    if i == 0:
        cols = table.max() <= 500_000
        cols = cols[cols].index
    table = table[cols]
    BASELINE_COLS = cols.copy()

    bar = sns.barplot(data=table, ax=axes[i],
                      #alpha=.5
                     )
    #swarm = sns.swarmplot(data=table, ax=axes[i], s=10, alpha=.85, edgecolor="
    axes[i].tick_params(rotation=90, labelsize=10, axis="x")
    axes[i].tick_params(labelsize=10, axis="y")
    axes[i].set_ylim(0, 500_000)
```

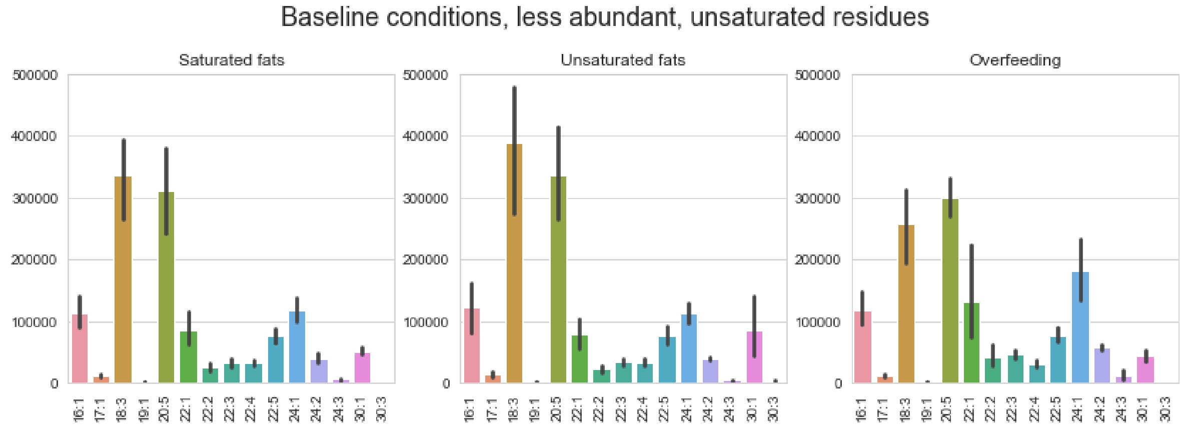

In [53]:

```
label = "After treatment, less abundant, unsaturated residues"

fig, axes = plt.subplots(1, 3, figsize=(14,4))
plt.suptitle(label, size=18, y=1.05)
labelled_columns = 2

for i, key in enumerate(("Saturated fats", "Unsaturated fats", "Overfeeding")):

    axes[i].set_title(f"{key}")

    #table = data.loc[baseline_samples.get(key), :].copy()
    table = data[data["diet"] == key].copy()

    # saturated or insaturated?
    # ==========================
    table = table.loc[:,[not saturated(x) and max_carbon(x, 30) for x in table._

    # the same as previous plot
    table = table[BASELINE_COLS]

    bar = sns.barplot(data=table, ax=axes[i],
                      #alpha=.5
                     )
    #swarm = sns.swarmplot(data=table, ax=axes[i], s=10, alpha=.85, edgecolor="
    axes[i].tick_params(rotation=90, labelsize=10, axis="x")
    axes[i].tick_params(labelsize=10, axis="y")
    axes[i].set_ylim(0, 500_000)
```

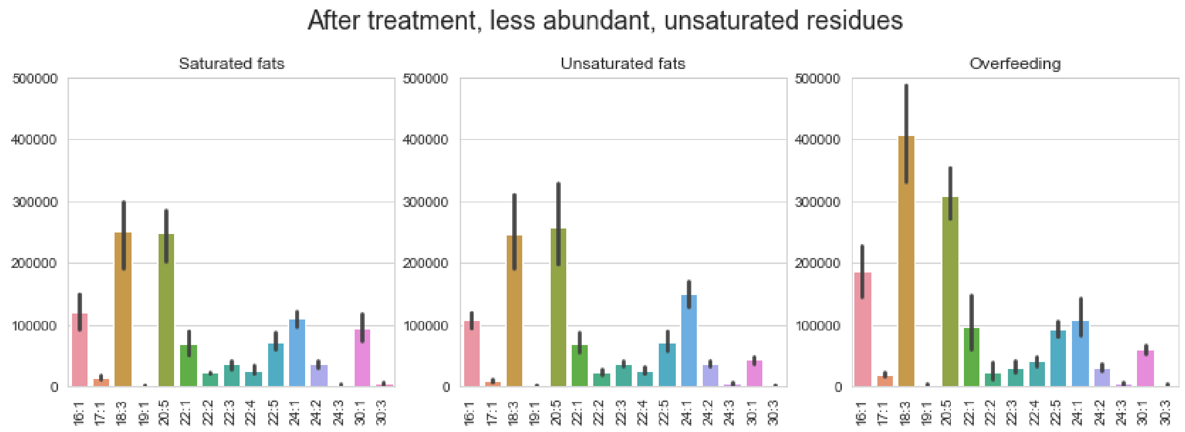

Supplement: Supplementary file 1 — Supplementary information 1 [file 41598_2020_70259_MOESM1_ESM.pdf]
