# Supplementary material for: liputils: a Python module to manage individual fatty acid moieties from complex lipids
Source: Sci Rep. 2020 Aug 7;10:13368. doi: 10.1038/s41598-020-70259-9 (PMC7415148; doi:10.1038/s41598-020-70259-9)
Supplement: Supplementary file 2 — Supplementary information 2 [file 41598_2020_70259_MOESM2_ESM.pdf]

Supplemental Material

liputils - a Python module to manage individual fatty acid moieties from complex lipids

Stefano Manzini[1*], Marco Busnelli[1*], Alice Colombo[1], Mostafa Kiamehr[2], Giulia Chiesa[1]

[1]Department of Pharmacological and Biomolecular Sciences
Università degli Studi di Milano,
20133 Milano, Italy

[2] Faculty of Medicine and Health Technology,
Tampere University,
33014 Tampere, Finland

* These authors equally contributed to this work.

Corresponding authors:
Giulia Chiesa, giulia.chiesa@unimi.it
Stefano Manzini stefano.manzini@unimi.it

Supplementary Figure S1

A

41598_2017_BFsrep44503_MOESM67_ESM.xls

	A	B	C	D	E	F	G
1		Group	Plpp3_WT	Plpp3_WT	Plpp3_KO	Plpp3_WT	Plpp3
2		Diet	western	western	western	western	west
3	Lipid class	Lipid name	16-1	16-11	16-12	16-2	16
4	CE	CE 14:0	337.62167	487.77843	457.52704	358.94604	488.6
5	CE	CE 15:0	201.57888	284.39835	244.04901	185.30819	285.5
6	CE	CE 16:0	6063.54769	8875.01807	7159.75140	5527.55985	9049.
7	CE	CE 16:1	2128.69699	4389.60990	3579.84433	3010.09907	3493.
8	CE	CE 17:0	838.04332	1066.96406	885.65583	757.92779	1052.
9	CE	CE 17:1	358.28467	512.73542	478.67850	407.68995	475.7
10	CE	CE 18:0	1535.53361	1859.99044	1598.96027	1178.68799	1807.
11	CE	CE 18:1	8720.33776	11175.62366	9223.33774	9949.22209	12983.
12	CE	CE 18:2	1479.38418	2299.69718	2088.33231	1875.98259	2071.
13	CE	CE 18:3	215.65419	354.54567	317.47278	256.70301	313.6
14	CE	CE 19:0	51.32035	100.10614	76.29747	46.02304	81.7
15	CE	CE 19:1	79.71011	104.21516	93.38671	78.67111	119.0
16	CE	CE 19:2	9.31680	15.70018	13.40323	16.71022	[illegible]

B

	16-1	16-11	16-12	16-2	16-3	16-4	
14:0	366.207151	519.080276	489.623404	391.997333	517.263376	394.195228	500.
15:0	201.578879	284.398350	244.049011	185.308187	285.598587	203.610721	256.
16:0	8719.581657	12477.578985	NaN	8855.969034	12123.860802	8722.796188	10544.
16:1	2263.292995	4661.111102	3825.726416	3188.963147	3638.123680	2995.194100	3463.
17:0	838.043317	1066.964064	885.655828	757.927793	1052.758402	789.159200	1014.
17:1	358.284673	512.735415	478.678504	407.689948	475.720716	372.042406	501.
18:0	2777.439159	3012.836369	2657.698155	2632.689125	3282.076417	2440.301592	3041.
18:1	10997.217033	14282.296976	11941.622887	13083.144031	15512.540700	12791.730402	14531.
18:2	2398.447524	NaN	NaN	NaN	NaN	2428.383267	3048.
18:3	216.516554	NaN	318.100907	257.375889	314.452189	218.063460	293.
19:0	51.320348	100.106145	76.297469	46.023043	81.708145	52.746490	64.
19:1	79.710113	104.215156	93.386714	78.671108	119.009738	80.840988	109.
19:2	9.316802	15.700177	13.403230	16.710221	25.750033	14.385706	11.
20:0	32.585812	59.859201	NaN	NaN	39.205712	35.475635	33.

Graphical visualization of tabular data. For a quick tutorial of how to use liputils to extract residue information from lipidomics data, the following table is used (**A**). Data is arranged with samples in column, with analytes in row. To be processed directly with liputils `make_residues_table()` function, a table needs to avoid column multi-indexing, and have lipids as row index. This table needs to be loaded in pandas by specifying to skip the first two rows and to index the second column, or needs prior editing and all unwanted columns and row removed.

In **B**, all lipid residues have ben counted per sample. NaN stands for “not a number”, and it is the way Numpy has to represent blank/missing values. When saved to a csv or Excel file, these values will result in empty cells. Missing values derive from residues that are present in the data, but are not represented in that particular sample.

Supplementary Figure S2

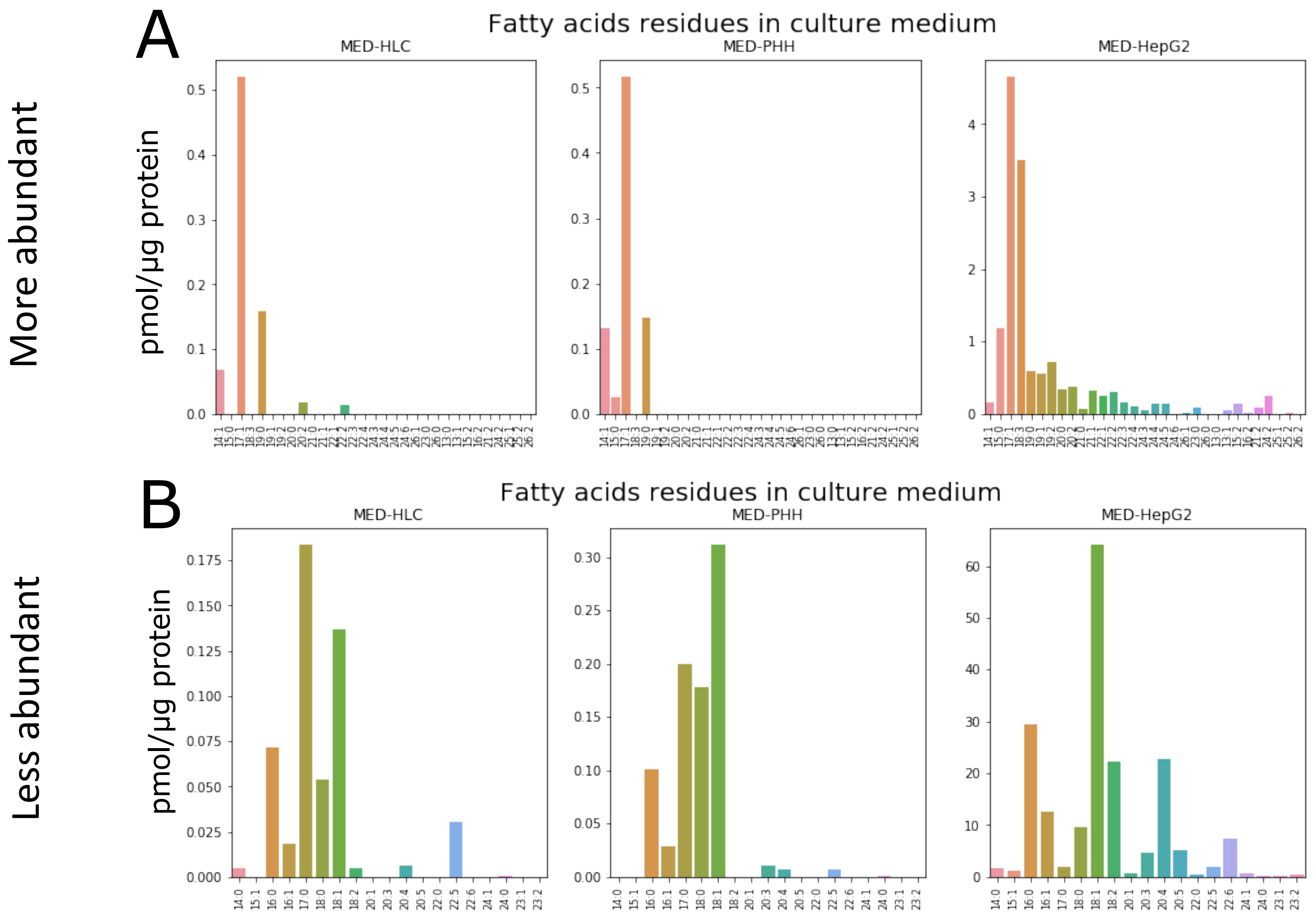

Fatty acid moieties extracted from stock media. Residues extracted from lipidomic data of HepG2, human hepatocyte-like cells (HLCs) and human primary hepatocytes (HPPs) culture media are shown (**A**-**B**) (n=1 per sample). HLCs and PHHs media were chemically defined, while HepG2 medium included 10% FBS comprised of complex lipid molecules. Units were unchanged during the extraction and are the same as the input data (pmol/µg protein).

Supplementary Figure S3

A

Download results as tab-delimited text

Input name	RefMet name	Formula	Mass	MW structure
PE(P-40:6)/PE(O-40:7)	PE(P-40:6)/PE(O-40:7)	C45H78NO7P	775.5516	

B

RefMet Compounds with Formula C45H78NO7P

Name	Formula	Mass
PC(P-37:6)/PC(O-37:7)	C45H78NO7P	775.551592
PE(P-16:0/24:6)/PE(O-16:1/24:6)	C45H78NO7P	775.551592
PE(P-18:0/22:6)/PE(O-18:1/22:6)	C45H78NO7P	775.5516
PE(P-18:1/22:5)/PE(O-18:2/22:5)	C45H78NO7P	775.5516
PE(P-20:1/20:5)/PE(O-20:2/20:5)	C45H78NO7P	775.551592
PE(P-40:6)/PE(O-40:7)	C45H78NO7P	775.5516

Graphical output of the online RefMet translator. The RefMet compound name translator, available at the following address: (https://www.metabolomicsworkbench.org/databases/refmet/name_to_refmet_form.php) attempts at translating non-RefMet compliant strings into official RefMet compounds (**A**). For low-resolution mass isomers, a list of possible molecular lipids that can be the parent compound is given (**B**).

Supplementary Table 1

Residue	Comparison	Difference	Lower	Upper	Adjusted p-value
14:0	HepG2-HLC-3	4.504313941	0.930846547	8.077781336	0.008694647
14:0	HepG2-HLC-4	4.765475723	1.136602429	8.394349017	0.005990598
14:0	HepG2-HLC-5	5.895734412	2.105499871	9.685968952	0.001038805
14:0	HepG2-PHH	8.171247305	3.794661441	12.54783317	0.000106378
14:0	HLC-3-PHH	3.666933364	0.093465969	7.240400759	0.042326626
14:0	HLC-4-PHH	3.405771582	-0.22310171	7.034644876	0.073199923
14:0	HLC-5-PHH	2.275512894	-1.51472165	6.065747434	0.413794388
14:1	HepG2-HLC-3	0.220805953	0.15990581	0.281706095	1.28435E-09
14:1	HepG2-HLC-4	0.256900038	0.195055652	0.318744425	8.0487E-11
14:1	HepG2-HLC-5	0.248820162	0.184225806	0.313414517	3.79583E-10
14:1	HepG2-PHH	0.291483518	0.216896381	0.366070655	2.83046E-10
14:1	HLC-3-PHH	0.070677565	0.009777423	0.131577707	0.01736879
15:2	HepG2-HLC-3	0.137304833	0.083570279	0.191039387	8.57222E-07
15:2	HepG2-HLC-4	0.155224912	0.100657215	0.209792609	1.30395E-07
15:2	HepG2-HLC-5	0.16128325	0.104289149	0.218277351	1.43257E-07
15:2	HepG2-PHH	0.169815569	0.10400445	0.235626688	7.2534E-07
18:1	HepG2-HLC-4	24.90752905	-20.9052291	70.72028722	0.510581085
18:1	HepG2-PHH	78.38682438	23.13455912	133.6390896	0.002810973
18:1	HLC-3-PHH	58.37080932	13.25752365	103.484095	0.006871231
18:1	HLC-4-PHH	53.47929533	7.66653716	99.2920535	0.016581797
18:1	HLC-5-PHH	43.47853545	-4.37132989	91.32840078	0.087584786
18:2	PHH-HepG2	26.67016862	21.63910102	31.70123622	4.61E-13
18:2	PHH-HLC-3	27.06354373	22.95569424	31.17139323	2.4E-14
18:2	PHH-HLC-4	28.73763907	24.56609819	32.90917995	2.2E-14
18:2	PHH-HLC-5	28.21689171	23.85985936	32.57392406	2.6E-14
19:1	HepG2-HLC-3	0.143283663	0.028781198	0.257786128	0.009269628
19:1	HepG2-HLC-4	0.160547234	0.044269432	0.276825037	0.003696694
19:1	HepG2-HLC-5	0.168075629	0.046627425	0.289523833	0.003612389
19:1	HepG2-PHH	0.225053276	0.084816969	0.365289583	0.00072411
20:1	HepG2-HLC-3	1.54129587	0.826598667	2.255993072	1.32953E-05
20:1	HepG2-HLC-4	1.605891514	0.880113074	2.331669954	8.95599E-06
20:1	HepG2-HLC-5	1.844624653	1.086573796	2.602675511	1.94759E-06
20:1	HepG2-PHH	2.205286886	1.329965153	3.080608619	1.08941E-06
20:4	PHH-HepG2	11.81816819	5.894196339	17.74214003	4.21323E-05
20:4	PHH-HLC-3	12.36240042	7.525497661	17.19930318	8.53755E-07
20:4	PHH-HLC-4	13.06167378	8.149775806	17.97357175	4.31178E-07
20:4	PHH-HLC-5	10.59595832	5.46564821	15.72626843	2.54284E-05
24:5	HepG2-HLC-3	0.210227209	0.076868649	0.343585769	0.000891533
24:5	HepG2-HLC-4	0.273972769	0.138546512	0.409399027	3.44339E-05
24:5	HepG2-HLC-5	0.23727735	0.095829237	0.378725463	0.000425134
24:5	HepG2-PHH	0.308441094	0.145110881	0.471771306	9.10419E-05
24:6	HepG2-HLC-3	0.45441577	0.319676332	0.589155208	5.34231E-09
24:6	HepG2-HLC-4	0.504574457	0.367745911	0.641403003	9.15416E-10
24:6	HepG2-HLC-5	0.475211758	0.332299002	0.618124513	7.02772E-09
24:6	HepG2-PHH	0.544408685	0.37938725	0.709430121	8.18578E-09

Detailed statistically significant differences for each comparison, per residue. Statistically significant differences were determined with ANOVA followed by Tukey’s post-hoc test (n=9 HLC-3, n=8 HLC-4, n=6 HLC-5, n=3 PHH and HepG2 samples).

Supplementary Table 2

Residue	Comparison	Difference	Lower	Upper	Adjusted p-value
18:3	Overfeeding-Saturated fats	83.0989348	32.55919384	133.6386758	0.001497514
20:1	Saturated fats-Overfeeding	69.95086247	21.33220413	118.5695208	0.004720554
20:1	Unsaturated fats-Overfeeding	79.98050715	31.36184881	128.5991655	0.001490594
20:2	Saturated fats-Overfeeding	37.06273322	1.541102354	72.58436408	0.040064848
20:2	Unsaturated fats-Overfeeding	37.24209302	1.720462156	72.76372389	0.039035724
20:3	Overfeeding-Saturated fats	46.92097803	1.782919941	92.05903611	0.040873888
20:3	Overfeeding-Unsaturated fats	46.96134996	1.82329187	92.09940804	0.040686328
20:4	Saturated fats-Overfeeding	23.63496846	3.364934857	43.90500207	0.02103841
20:4	Unsaturated fats-Overfeeding	31.10317186	10.83313825	51.37320547	0.002767359
20:5	Overfeeding-Saturated fats	22.77345043	0.33089319	45.21600767	0.046393858
20:5	Overfeeding-Unsaturated fats	26.9670954	4.524538156	49.40965264	0.017378353
22:2	Unsaturated fats-Overfeeding	66.11189494	10.99301677	121.2307731	0.01758215
22:3	Saturated fats-Overfeeding	60.9218937	2.183451685	119.6603357	0.041365586
22:4	Overfeeding-Saturated fats	56.98806747	4.495712921	109.480422	0.032177736
22:6	Saturated fats-Overfeeding	29.37200864	11.42672197	47.31729532	0.001561442
22:6	Unsaturated fats-Overfeeding	22.6405984	4.695311727	40.58588508	0.012548342
24:1	Saturated fats-Overfeeding	39.81046071	12.01919479	67.60172662	0.004888639
24:1	Unsaturated fats-Overfeeding	66.73494923	38.94368331	94.52621514	2.47704E-05
26:0	Saturated fats-Unsaturated fats	384.6559913	60.23615386	709.0758288	0.018933907
30:1	Saturated fats-Unsaturated fats	92.89024137	8.0615839	177.7188988	0.030635146

Detailed statistically significant differences for each comparison, per residue. Statistically significant differences were determined with ANOVA followed by Tukey's post-hoc test (n=7 subjects per group).

Supplemental Materials and Methods

General description. We have developed `liputils` as a lightweight Python library focused on text-based recognition of lipid identifiers that can be seamlessly integrated in a Python-based data analysis pipeline. Its primary function is to provide fatty acid moieties information from any RefMet-compliant lipid annotation.

An in-depth, Python-based analysis of a dataset is available as Supplemental analysis protocol online in PDF form, or in its native Jupyter notebook file format at the following address.

In the main text, the User is presented with a basic protocol that automatically processes lipidomics data in tabular format in order to extract residue information and package the results into a new, easily readable data table. In here, we will be discussing some useful functions and methods of the library that can be exploited by the more advanced User and suited to personalized analysis pipelines.

Functionality of the `Lipid` class. In `liputils`, every lipid identifier (a text string) is managed through the `Lipid` class. Then, lipid properties can be accessed through each object's methods. An example:

```
>>> from liputils import Lipid

>>> lip = Lipid("Cer(d20:0/22:0)")

>>> lip.name
'Cer(d20:0/22:0)'

>>> lip.refmet_class()
'Cer'
```

`Lipid.name` is produced by the class constructor. The vast majority of other methods are callable, in order to save time when initializing each new `Lipid` object. In this case, `Lipid.refmet_class()` is a method that computes the lipid class, given as abbreviation, upon call. If needed, the `amount` parameter can be used to define the amount of the lipid being processed (it defaults to picomoles), and to calculate the number of molecules:

```
>>> lip = Lipid("Cer(d20:0/22:0)", amount=0.12512)
```

```
>>> lip.molecules
75349026402.78401
```

Other units can be specified:

```
>>> lip = Lipid("Cer(d20:0/22:0)", amount=0.12512, unit="attomoles")

>>> lip.molecules
75349.02640278402
```

Fatty moieties identification. The backbone of the `Lipid` class functionality is the ability of decoding from lipid identifiers their carbon chains composition. `liputils` accepts a generic lipid format in the form of `CLASS N:N/M:M/../..` or `CLASS(N:N/M:M/../..)(other mass isobars)`, or [fully RefMet-compliant residue naming](). If unsure about your data format, you can try and batch-translate your lipid IDs with RefMet's online translator. By adhering to RefMet's nomenclature, any compliant lipid name will be properly managed by liputils's method `Lipid.refmet_residues()`.

```
>>> lip1 = Lipid("octadecatrienoic acid")

>>> lip2 = Lipid("linolenic acid")

>>> lip3 = Lipid("FA(18:3)")

>>> lip4 = Lipid("linoleyl palmitate")

>>> lip1.refmet_residues()
(['18:3'], 1)

>>> lip2.refmet_residues()
(['18:3'], 1)

>>> lip3.refmet_residues()
(['18:3'], 1)

>>> lip4.refmet_residues()
(['18:1', '16:0'], 1)
```

The method returns a `list` of residues that were found in the lipid identifier, and an integer index that refers to the *ambiguities* found within the lipid identifiers. This feature was introduced to let the User choose what to do in the presence of unresolved mass isobars.

For example, in the case of this non-RefMet compliant lipid identifier taken from a commercial lipidomic analysis:

```
>>> lip = Lipid("TAG 48:2 total (14:0/16:0/18:2)(14:0/16:1/18:1)(16:0/16:1/16:1)")

>>> lip.refmet_residues()
(['48:2', '14:0', '16:0', '18:2', '14:0', '16:1', '18:1', '16:0', '16:1', '16:1'],
3)
```

In this case, `liputils` reports each residue in the `list` it returns, but it also returns `3` as ambiguity index, to reflect the fact that the molecular lipid was not fully resolved, and there were three different but equivalent mass isobars.

When using non-Refmet compliant lipids, Users are encouraged to use `Lipid.lipid_class()` and `Lipid.residues()` methods instead:

```
>>> lip.refmet_class() # not appropriate
'TAG 48:2 total'

>>> lip.lipid_class() # appropriate
'TAG'

>>> lip.residues() # same result, in this case
(['14:0', '16:0', '18:2', '14:0', '16:1', '18:1', '16:0', '16:1', '16:1'],
3)
```

If desired, it can be useful to suppress all unresolved mass isobars by setting the `drop_ambiguous` parameter to `True` (it defaults to `False`):

```
>>> lip.residues(drop_ambiguous=True)
([], 0)
```

In this case, an empty `list` is returned together with `0` ambiguity index.

Helper functions. `liputils` contains some functions that complement the `Lipid` `class` functionality. In the manuscript (**Materials and Methods → Procedure**), the basic use case of `make_residues_table()` is presented. There are parameters that can be set to alter the function's default behavior:

`drop_ambiguous`: defaults to `False`. As in the case mentioned above, this produces a residues table that does not take into account unresolved mass isobars.

`name`: as `make_residues_table()` returns a nameless `pandas.DataFrame` object, this parameters sets a `string` attribute within the object, under `pandas.DataFrame.name`, that can be useful when a string identifier for exporting the table to a `.csv` file (or for otherwise any other use), especially when working with multiple objects simultaneously. Defaults to `"residues_table"`.

`replace_nan`: this replaces any missing value (empty cells) in the input table with the desired value. It defaults to `0`.

`cleanup`: this parameter is used to avoid processing some lipids that may be found in the data but are not meaningful to process. These may include, for example, identifiers with "total" (like "total cholesterol"), or abbreviations thereof (like "TC" for total cholesterol, or "FC" for free cholesterol). The list of unwanted terms is read from another parameter, `unwanted` (see below). It defaults to `True`.

`unwanted`: this parameter defaults to a list of unwanted identifiers, that are altogether skipped in the processing if cleanup is `True` (the default behavior). This parameter can be replaced by a User-defined `list` of choice. It defaults to `["total", "fc", "tc"]`.

`absolute_amount`: this switches the function from counting the actual residues amount, in the same unit as in the input table, to counting in the actual number of residues. For example, if the input table is in picomoles/µg tissue, by default the table produced by `make_residues_table()` will be in picomoles/µg tissue. Otherwise, if `absolute_amount` is set to True, then the actual number of residues will be counted. The default `unit` is `"picomoles"`, but this can be set otherwise by passing another `unit` as keyword argument. It defaults to `False`.

In addition to the use case presented in the manuscript, an additional example can be found in the Supplemental analysis protocol at page 5.

Two other functions come handy when processing residues subsets. To focus on particular residues, it is possible to mix `saturated()` and `max_carbon()` to dictate which residues to keep and which to discard. Specifically:

`saturated()`: its intended use is with string residue identifiers (in the form of `\d:\d`). It returns `True` if the residue contains unsaturations, `False` otherwise.

`max_carbon()`: its intended use is with string residue identifiers (in the form of `\d:\d`). An integer number of desired carbon atoms must also be specified when calling the function. If

the number of carbon atoms in the residue is lower or equal to the specified maximum allowed value, the function returns `True`, `False` otherwise.

Some examples:

```
>>> from liputils import saturated, max_carbon

>>> saturated("12:0")
True

>>> saturated("12:1")
False

>>> max_carbon("12:1", 11)
False

>>> max_carbon("12:1", 12)
True

>>> max_carbon("12:1", 13)
True
```

These functions come handy in *list comprehensions* (especially useful when the result is then used to exclude/include elements in an index):

```
>>> my_residues = ["12:0", "17:1", "24:0", "24:1", "24:2", "26:3"]

>>> [not saturated(x) and max_carbon(x, 24) for x in my_lipids]
[False, True, False, True, True, False]
```

Conveniently, `saturated()` can be negated via the `not` operator if the residue is required to having exactly zero unsaturations.

Example use of these functions can be found in the Supplemental analysis protocol, starting from page 16, when they are used to restrict the plots to specific residues.

Online Documentation. As `liputils` is in active development, the online documentation reflects the latest changes and updates to the library. It can be accessed at [`liputil`'s pip package manager page,]() or in [`liputil`'s GitHub repository]().
